# Oleic Acid Integrated Acetalated Dextran Nanoparticles for Enhanced Chemotherapeutic Delivery to the Bone Marrow

**DOI:** 10.1021/acsami.5c16936

**Published:** 2025-10-14

**Authors:** Krystal A. Hughes, William H. Pentz, Bishal Misra, Morgan Surface, Werner J. Geldenhuys, Salik Hussain, Sharan Bobbala

**Affiliations:** † Department of Pharmaceutical Sciences, West Virginia University School of Pharmacy, Morgantown, West Virginia 26505, United States; ‡ School of Medicine, 5631 West Virginia University, Morgantown, West Virginia 26506, United States; § Department of Clinical Pharmacy, West Virginia University School of Pharmacy, Morgantown, West Virginia 26505, United States; ∥ Department of Neuroscience, West Virginia University School of Medicine, Morgantown, West Virginia 26505, United States; ⊥ Department of Microbiology, Immunology and Cell Biology, West Virginia University School of Medicine, Morgantown, West Virginia 26505, United States; # Department of Physiology, Pharmacology and Toxicology, West Virginia University, Morgantown, West Virginia 26505, United States

**Keywords:** bone marrow, passive accumulation, oleic acid, dextran, polymer−lipid hybrid, hematology

## Abstract

Bone marrow targeted delivery of chemotherapeutics remains critical in the treatment of hematological malignancies such as acute lymphoblastic leukemia (ALL). Current drug delivery platforms to treat ALL do not specialize in enhancing drug accumulation in the bone marrow, often leading to suboptimal therapeutic responses and off-target side effects. Here, we developed a polymeric lipid hybrid nanoparticle (PLHP) platform through integration of a pH-responsive acetalated dextran (Ac-Dex) polymer and oleic acid (OA), an endogenous bone marrow-rich fatty acid, for enhanced accumulation of therapeutic payloads to bone marrow. PLHPs formulated using the flash nanoprecipitation were monodisperse with sizes below 200 nm and allowed encapsulation of diverse payloads. Of note, lyophilized PHLPs retained greater than 80% of encapsulated payloads following rehydration. Confocal imaging confirmed precise intracellular release of hydrophilic and hydrophobic payloads in B-cell ALL cell lines. In healthy BALB/c mice and B-cell ALL bearing NSG mice, we demonstrate that incorporation of OA into the Ac-Dex nanoparticles enhances payload accumulation in the femur and tibia compared to non-OA containing nanoparticles. In the B-cell ALL disease model, vincristine encapsulated into Ac-Dex OA nanoparticles significantly improved the survival of mice while preserving locomotor function, and mitigated terminal weight loss as compared to the systemically administered free drug and blank nanoparticles. Together, these findings show the promising translational potential of oleic acid integrated Ac-Dex nanoparticles for clinical use in bone marrow-derived diseases.

## Graphical abstract

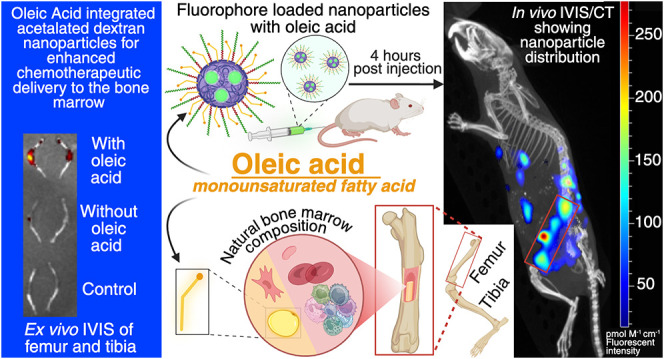

## 1 Introduction

Nanoparticles are the dominant advanced drug delivery platforms that allow precise delivery of physicochemically diverse therapeutics to the target sites of interest by overcoming formulation and biological barriers.^1–3^ Lipid, polymeric, and polymeric lipid hybrid nanoparticles (PLHPs) are among the frontrunners in nanoparticle delivery platforms for biomedical applications due to their biocompatible and biodegradable nature.^4,5^ Specifically, PLHPs are currently of great interest due to their superior biomimetic attributes and mechanical stability, which are attained from the polymeric component and lipid component, respectively.^6–9^ Although PLHPs are an advancement in nanotechnology, the choice of polymer/lipid, utilization of nontoxic solvents in the fabrication process, payload retention, drug release kinetics, and interactions with biological barriers are deemed critical for their success.^10–12^ Here, we chose acetalated dextran (Ac-Dex) as the polymer component due to its versatility in biocompatibility and pH-responsive nature.^4,13^ We have previously shown that Ac-Dex nanoparticles fabricated using a nontoxic solvent ethanol, and PEGylated surfactant d-α-tocopherol poly­(ethylene glycol) 1000 succinate (TPGS) as the stabilizer achieve high particle stability and encapsulation of payloads. Due to their pH-responsive nature, Ac-Dex nanoparticles minimize the leakage of payloads in physiological pH, allowing precise release of payloads in acidic pH, such as cellular endolysosomal conditions, which can be critical in the intracellular delivery of chemotherapeutics.^13^

Intentional accumulation and retention of nanoparticle encapsulated payloads in tissues such as bone marrow without the use of complex surface functionalization or targeting approaches remains challenging.^14,15^ The bone marrow, for example, possesses sinusoids with discontinuous fenestrations ranging from 100–200 nm in the endothelial layers, which may aid in the nanoparticle delivery process.^16^ However, the kinetics of nanoparticle retention inside the bone marrow and how different nanoparticle compositions will impact the delivery efficiency of therapeutics in the bone marrow microenvironment are often understudied. Here, we studied Ac-Dex nanoparticles integrated with oleic acid (OA), an endogenous unsaturated fatty acid that naturally occurs within the bone marrow microenvironment for nanoparticle retention and delivery of a functional payload. Of note, OA is found within the bone marrow, specifically adipose tissue, and it is released into the microenvironment as part of the lipid metabolic pathways.^17^ Fatty acids, such as palmitic, oleic, and lauric acids, are essential components of the bone marrow. These fatty acids can be utilized as an energy source for bone remodeling and hematopoietic cell regulation.^18^

The bone marrow can be divided into red and yellow marrow, each with a distinct composition and function. The red bone marrow is responsible for hematopoiesis, a differentiation process that produces blood cells and begins with hematopoietic stem cells (HSC).^19^ The HSCs and other progenitor cells utilize endogenous fatty acids as an energy source and promoter for differentiation.^20^ The yellow marrow primarily comprises adipocytes and is considered lipid-rich, which secretes the necessary fatty acids for the red marrow, which only contains 20–40% adipocytes.^21^ Integrating OA into a nanoparticle delivery system may lead to interaction with the bone marrow OA pathways, enhancing passive accumulation and local retention of payloads. Bone marrow accumulation of a nanoparticle could be highly impactful for treating hematological malignancies. Acute lymphoblastic leukemia (ALL) is one example of a hematological malignancy that could benefit from novel drug delivery. ALL is caused by abnormal lymphocyte production and overabundance within the bone marrow. Initial remission following chemotherapy is often achievable; however, recurrence is common, and the subsequent chemotherapeutic resistance leads to poor prognoses. This calls for an urgent need to develop novel therapeutic strategies that can precisely deliver small-molecule therapeutics to the leukemic cells and the bone marrow microenvironment, which may help overcome drug-resistant mechanisms.^22^

This study presents the fabrication and *in vitro* and *in vivo* characterization of an Ac-Dex OA nanoparticle platform that can achieve passive accumulation in the bone marrow, which may be utilized to help overcome the treatment gap of current leukemia therapeutics. In a B-cell ALL human xenograft mouse model, we further demonstrated improved antileukemic activity and reduced peripheral neurotoxicity of vincristine following delivery via Ac-Dex OA nanoparticles. This delivery platform holds a great promise to be utilized for the treatment of hematological cancers and bone marrow metastases.

## 2 Results and Discussion

### 2.1 Fabrication and Physicochemical Characterization of Ac-Dex TPGS OA Nanoparticles

We previously optimized Ac-Dex TPGS nanoparticles using the flash nanoprecipitation (FNP) approach and ethanol as a nontoxic organic solvent.^13^ In this study, we modified Ac-Dex nanoparticles by adding a lipid component into the formulation to form a polymeric lipid hybrid nanoparticle. This nanoparticle formulation consists of Ac-Dex, a pH-responsive polymer; TPGS, a PEGylated nonionic surfactant; and OA, an unsaturated fatty acid lipid, as seen in Figure 1A. We propose that the likely structural architecture, as seen in Figure 1B, includes Ac-Dex as the hydrophobic polymeric core. TPGS is a PEGylated vitamin E derivative that stabilizes the polymeric core and prolongs the circulation time of nanoparticles. OA is an amphiphile with a hydrophobic fatty acid tail and a hydrophilic carboxylic acid headgroup. We hypothesize that the hydrophobic tail is intercalated on the Ac-Dex core, and the hydrophilic carboxylic acid head groups are oriented to face the aqueous environment, similarly to the PEG 1000 component of TPGS.

**Figure 1:**
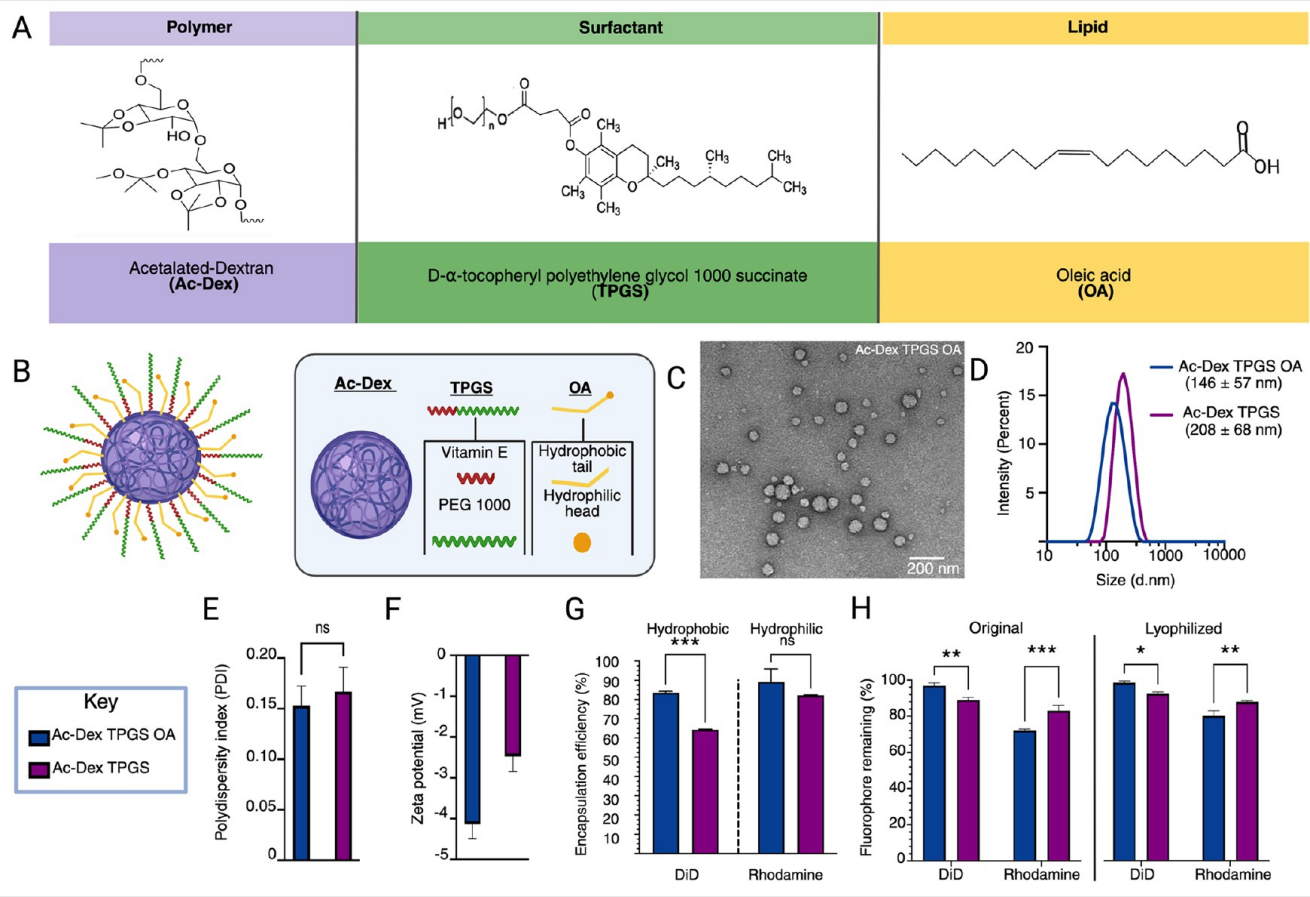
Physicochemical characterization of polymeric lipid hybrid nanoparticle, Ac-Dex TPGS OA, and polymeric nanoparticle Ac-Dex TPGS. (A) Chemical structures of each component incorporated into the hybrid particle, (B) Schematic of the proposed nanoparticle architectural structure, (C) Transmission electron microscopy (TEM) image of Ac-Dex TPGS OA (Scale bar = 200 nm), (D) Size characterization through dynamic light scattering (DLS) reported as intensity percent, (E) Polydispersity index (PDI) obtained through DLS with no significant differences between Ac-Dex TPGS OA and Ac-Dex TPGS as determined by an unpaired *t*-test, (F) Zeta potential obtained through electrophoretic light scattering (ELS), statistical significance was determined by an unpaired *t*-test (**p < 0.005) with data reported as mean ± s.d. (*n* = 3), (G) Encapsulation efficiency (EE%) determined for hydrophobic and hydrophilic compounds with statistical significance determined by two way ANOVA with šídák’s multiple comparison test (***p < 0.0005) with data reported as mean ± s.d. (*n* = 3), (H) Fluorophore retention determined for hydrophilic and hydrophobic compounds after lyophilization with 10% w/v mannitol, statistical significance was determined by two way ANOVA with šídák’s multiple comparison test (*p < 0.05, **p < 0.005) with data reported as mean ± s.d. (*n* = 3).

FNP allows rapid fabrication of lipid and polymeric nanoparticles while controlling particle size, distribution, encapsulation efficiency, and morphology.^23,24^ Regarding a multifunctional system such as PLHPs, FNP is a promising formulation method due to the decreased risk of aggregation and increased nanoparticle homogeneity by rapid millisecond mixing, which can encourage a uniform coassembly of the formulation components. Of note, a simple mixing process in a confined jet mixer using aqueous and organic solvents makes it ideal for optimizing polymer-to-lipid ratios during PLHN development. Here, we optimized Ac-Dex TPGS OA nanoparticles using 20 mg Ac-Dex and varying ratios of TPGS and OA (Table S1). When Ac-Dex particles were formulated with OA alone, particle stabilization was not sufficient; this is demonstrated by a polydispersity index (PDI) of 1.8, indicating large heterogeneity in particle size, which is further supported by the micron scale of the particles (4239 ± 1650 nm). However, formulations with TPGS, either alone or in combination with OA, were monodispersed and <200 nm. Particles with only TPGS had the smallest PDI at 0.04. This highlights the critical need for TPGS in particle stability, as evidenced by a slight increase in PDI to 0.15 when OA is combined with TPGS (Table S2). This PDI is still within limits to be considered monodisperse. During the formulation optimization process, a range of OA concentrations were tested, and we found that 2.5 mg oleic acid with 2.5 mg TPGS yielded nanoparticles with high monodispersity, which is evident through the polydispersity index of less than 0.3 (Table S1). The OA retention on the nanoparticle was found to be 21% (Figure S1). Transmission electron microscopy (TEM) confirmed the spherical morphology of Ac-Dex TPGS OA nanoparticles (Figure 1C). In addition, Ac-Dex TPGS nanoparticles with and without OA demonstrated similar morphological features in TEM (Figure S2). Dynamic light scattering (DLS) analysis revealed that the average particle size for Ac-Dex TPGS OA was below 200 nm and slightly smaller in comparison to Ac-Dex TPGS, as shown in Figure 1D. This size reduction may be due to multiple OA characteristics, which include its unsaturated nature and the hydrophobic carbon tail (18C). Oleic acid has previously been demonstrated to decrease nanoparticle size and reduce aggregation due to its unsaturated single–double bond backbone.^25^ The monounsaturated double bond allows OA flexibility that may lead to saturation on the particle surface with dense surface packing, with decreased aggregation. This may also contribute to the relatively low OA retention rate. Incorporation of OA did not impact the homogeneity of the size distribution, and Ac-Dex TPGS nanoparticles with and without OA were monodisperse with a polydispersity index below 0.3 (Figure 1E). Surface charge characterization through electrophoretic light scattering (ELS) reveals that Ac-Dex TPGS nanoparticles with and without OA possess a neutral surface charge (Figure 1F) with zeta potential ranging between −2.5 and −5 mV. These results could be mainly attributed to the presence of TPGS, a nonionic PEGylated surfactant on the surface of nanoparticles. OA, however, possesses a carboxylic acid head, which would likely be deprotonated at the neutral physiological pH, leading to a negative surface charge.^26^ Therefore, Ac-Dex TPGS OA exhibited slightly more negative surface charge than Ac-Dex TPGS. However, particles with a surface charge between −10 and +10 mV are often considered neutral.^27^

The encapsulation efficiency (EE%) was then evaluated for a hydrophobic (DiD dye) and hydrophilic (rhodamine dye) model fluorophores. Rhodamine was encapsulated with >80% efficiency, and no significant difference in percentage encapsulation efficiency was observed between Ac-Dex TPGS and Ac-Dex TPGS OA. The encapsulation efficiency of DiD was significantly higher in OA-containing nanoparticles with 83% as compared to Ac-Dex TPGS nanoparticles with 64% encapsulation (Figure 1G). This could be mainly due to the higher hydrophobic interactions associated with OA formulations during the nanoparticle fabrication.

The suitability of Ac-Dex TPGS OA nanoparticles for lyophilization was evaluated using different concentrations of widely studied cryoprotectants, including sucrose, mannitol, and trehalose (Figure S3). We found that 10% w/v of mannitol retained the size characteristics of Ac-Dex TPGS OA nanoparticles after lyophilization, comparable to prelyophilization. This is analogous to what our lab previously demonstrated with Ac-Dex TPGS nanoparticles.^13^ In addition to size, we studied the payload retention stability of Ac-Dex TPGS OA nanoparticles compared to Ac-Dex TPGS nanoparticles. Nanoparticles naturally diffuse payloads into the aqueous buffer inadvertently over time during storage, making their usage limited in maintaining therapeutic efficacy while decreasing unwanted, off-target effects. Specifically, hydrophilic compounds prone to faster diffusion and their retention pose a huge challenge during nanoparticle storage. Therefore, for long-term storage, payload retention remains critical following postlyophilization. As seen in Figure 1H, Ac-Dex TPGS OA nanoparticles retained 98% and 72% of the encapsulated hydrophobic and hydrophilic payloads, respectively.

The acetal group of Ac-Dex hydrolyze at acidic pH, resulting in the hydrophobic Ac-Dex converting back to the hydrophilic dextran.^13,28^ Our previous work demonstrated the pH-responsiveness of Ac-Dex TPGS nanoparticles, with complete dissolution of the nanoparticles and release of payloads over time in acidic conditions. To confirm whether OA containing Ac-Dex TPGS nanoparticles allows pH-responsive release of payloads, we incubated nanoparticles in acidic pH 4 and physiologic pH 7.4 buffers for up to 24 h (Figure S4). While the pH 4 group showed a slight increase in release, no statistically significant differences were observed at any time point measured compared to the pH 7.4 group. This suggests that the addition of OA might have enhanced particle stability, thereby reducing the degree of pH-responsive degradation of the particle at acidic pH. Interestingly, Ac-Dex TPGS OA nanoparticles retained 66% of the encapsulated payload after 24 h incubation in PBS buffer containing 10% fetal bovine serum (FBS), which mimics protein-rich physiological conditions (Figure S5). These results show the efficient stability of Ac-Dex TPGS OA nanoparticles in physiological conditions.

### 2.2 Effect of Various Lipid Components on *In Vivo* Biodistribution

Incorporation of lipids into nanoparticles can impact their structural characteristics and *in vivo* biodistribution.^29^ When selecting which lipids to screen for passive bone marrow accumulation, the endogenous composition of the bone marrow was taken into consideration (Figure 2D). The bone marrow is rich in free fatty acids, specifically in the yellow marrow, where adipocytes and mesenchymal stem cells reside.^30^ In the bone marrow, cellular uptake of long-chain fatty acids for metabolic processes occurs within the yellow marrow.^31,30^ In the yellow marrow, saturated fatty acids (lauric, myristic, palmitic, and stearic acid), in addition to unsaturated fatty acids (oleic and linoleic acid), are endogenously used. We utilized oleic and palmitic acid for formulation development because they are found in human bone marrow aspirates at high concentrations.^32^ OA is found in higher concentrations within the bone marrow supernatant fluid compared to systemic circulation.^17^ We hypothesize that Ac-Dex TPGS nanoparticles formulated with prominent endogenous fatty acids will demonstrate selective accumulation in the bone marrow due to increased lipid affinity.

**Figure 2:**
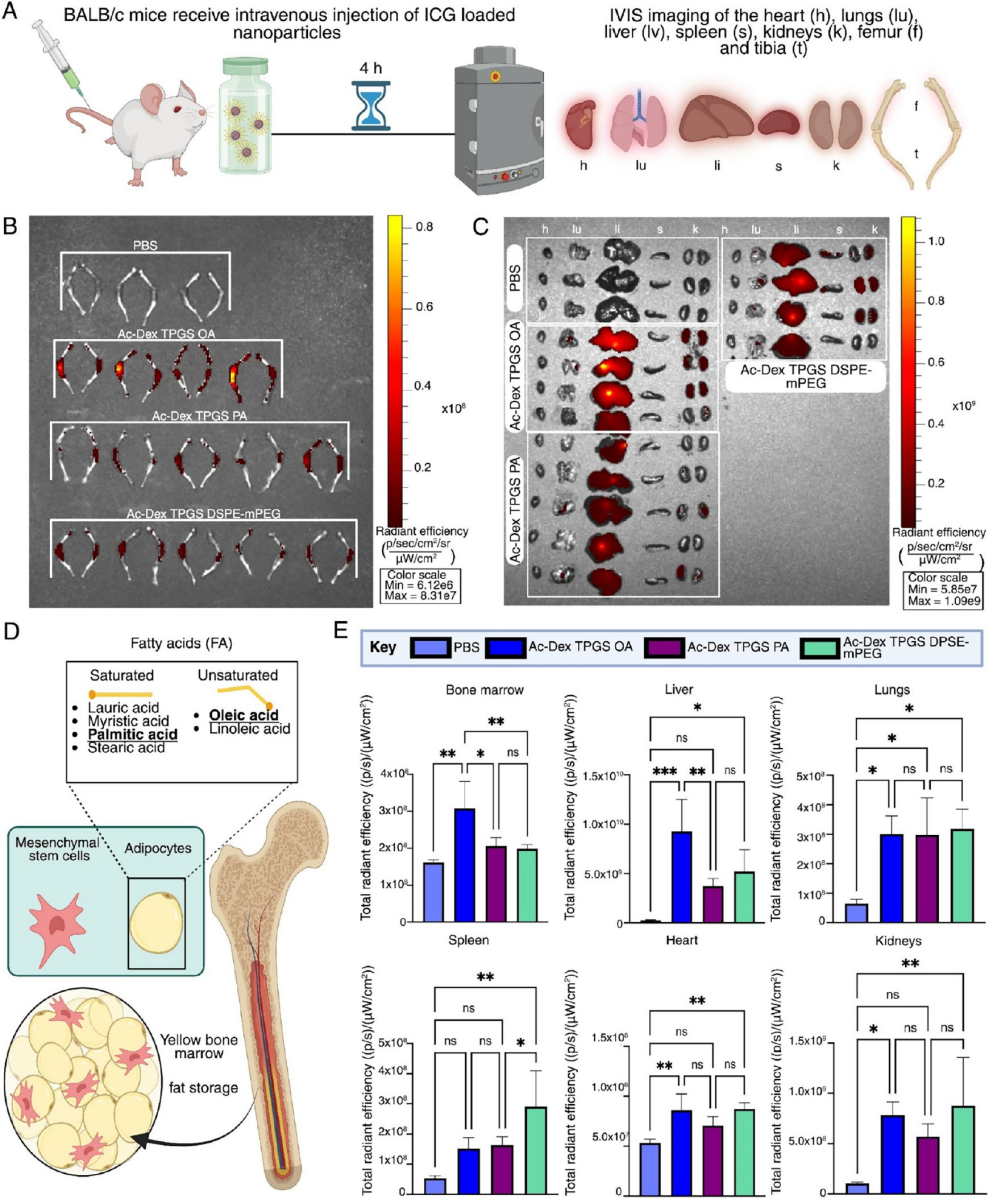
Lipid selection alters *in vivo* biodistribution of polymer lipid hybrid nanoparticles with oleic acid, promoting bone marrow accumulation. (A) Schematic of study design depicting intravenous administration of ICG-loaded Ac-Dex TPGS nanoparticles formulated with either oleic acid (OA), palmitic acid (PA), or DSPE-mPEG, followed by IVIS imaging. (B) IVIS imaging comparing hindlimb (femur and tibia) accumulation of nanoparticles containing OA (*n* = 4), PA *(n =* 5), and DSPE-mPEG *(n =* 5), to a PBS control *(n =* 3). (C) IVIS imaging of the heart (h), lungs (lu), liver (li), spleen (s), and kidneys (k) (*n* = 3–5 per group). (D) Schematic of endogenous bone marrow composition highlighting the saturated and unsaturated free fatty acids found within the yellow marrow. (E) Quantification of total radiant efficiency for the hindlimbs, as well as each organ. Statistical significance was determined by one way ANOVA with Tukey’s multiple comparison test (*p < 0.05, **p < 0.005, ***p < 0.0005) with data re*p*orted as mean ± s.d. (*n* = 3–5 per group).

Here, we formulated Ac-Dex TPGS nanoparticles with oleic acid (OA), palmitic acid (PA), or 1,2-Distearoyl-*sn*-glycero-3-phosphoethanolamine-*N*-[methoxy­(poly­(ethylene glycol)] (DSPE-mPEG). We chose DSPE-mPEG, C18-fatty acid, as a nonspecific bone marrow lipid, which is also commonly employed in clinically relevant liposome formulations.^33^ The nanoparticles were formulated with 20 mg Ac-Dex and 2.5 mg TPGS, in addition to 2.5 mg of the respective lipid. All nanoparticles were comparable in size (below 200 nm) (Figure S6) and encapsulation efficiency (EE%) of indocyanine green (ICG), a near-infrared fluorophore (Figure S7). ICG-loaded nanoparticles were intravenously injected in BALB/c mice and imaged *ex vivo* to observe organ-level biodistribution 4 h after injection (Figure 2A). The tissues that were harvested for *in vivo* imaging system (IVIS) imaging included the femur, tibia (Figure 2B), heart, lungs, liver, spleen, and kidneys (Figure 2C). Bone marrow accumulation was quantified by imaging the femur and tibia together, which are prominent sites for hematopoiesis.^34,35^ ICG signal represented as total radiant efficiency within the bone was significantly higher with the OA containing Ac-Dex TPGS nanoparticles as compared to PA or DSPE-mPEG containing Ac-Dex TPGS nanoparticles. Of note, there was no significant difference in signal between PA and DSPE-mPEG formulations, which suggests that the lipid component used in Ac-Dex TPGS nanoparticles can impact passive, but selective, biodistribution.

To confirm that the accumulation in the bone marrow was driven by the lipid component and not any inherent properties of Ac-Dex TPGS, we compared the biodistribution of ICG-loaded Ac-Dex TPGS nanoparticles with and without OA. Mice injected with ICG nanoparticles without the lipid component showed minimal hindlimb signal of live mice following IVIS imaging. However, the mice that received the formulation containing OA exhibited a prominent signal that is localized to the femur and tibia (Figure S8). Furthermore, we performed cellular-level biodistribution on bone marrow aspirates to analyze the uptake of Ac-Dex TPGS OA nanoparticles by resident bone marrow immune cells. (Figure S9). We found that uptake was broadly distributed between myeloid and lymphoid cell types, which is promising for hematologically focused delivery vehicles.

The mononuclear phagocytic system (MPS) is responsible for nanoparticle clearance and includes filtering organs such as the liver, spleen, kidneys, and lungs.^36,37^ Total radiant efficiency of whole organs revealed that Ac-Dex TPGS nanoparticles containing OA or PA, or DSPE-mPEG formulations were mainly distributed in the liver and the spleen. Notably, OA nanoparticles demonstrated significantly higher hepatic accumulation compared to the PA and DSPE-mPEG nanoparticles formulations (Figure 2E). Lung and kidney accumulation of all nanoparticles was higher than PBS, but did not vary significantly between formulations, regardless of the lipid component. These results are in agreement with our previously reported work on whole organ biodistribution studies of Ac-Dex TPGS nanoparticles.^13^ Overall, these results suggest preferential accumulation of Ac-Dex TPGS nanoparticles into bone marrow with incorporation of oleic acid.

### 2.3 Time-Dependent Biodistribution of Ac-Dex TPGS OA Nanoparticles

To characterize the accumulation of Ac-Dex TPGS OA nanoparticles over time in different organs, we intravenously injected ICG-loaded nanoparticles and collected organs at 2, 4, 8, and 12 h postinjection. The hindlimb bones (femur and tibia), heart, lungs, spleen, liver, kidneys, and serum were collected for IVIS imaging (Figure 3A). IVIS images demonstrate rapid accumulation of Ac-Dex TPGS OA nanoparticles within the hindlimbs (femur and tibia) at 2 h, which remained elevated until 4 h (Figure 3B). Of note, the total radiant efficiency between 2 and 4 h time points remains insignificant (Figure 3E). After 8 h, the ICG signal diminished and was no longer statistically significant compared to the PBS controls. A similar trend of higher accumulation in major organs until 4 h, followed by a significant reduction in ICG signal by 8 h, was observed (Figure 3C,E). These results were further strengthened by systemic clearance of Ac-Dex TPGS OA nanoparticles, represented by the ICG signal, and total radiant efficiency measured in serum (Figure 3D).

**Figure 3:**
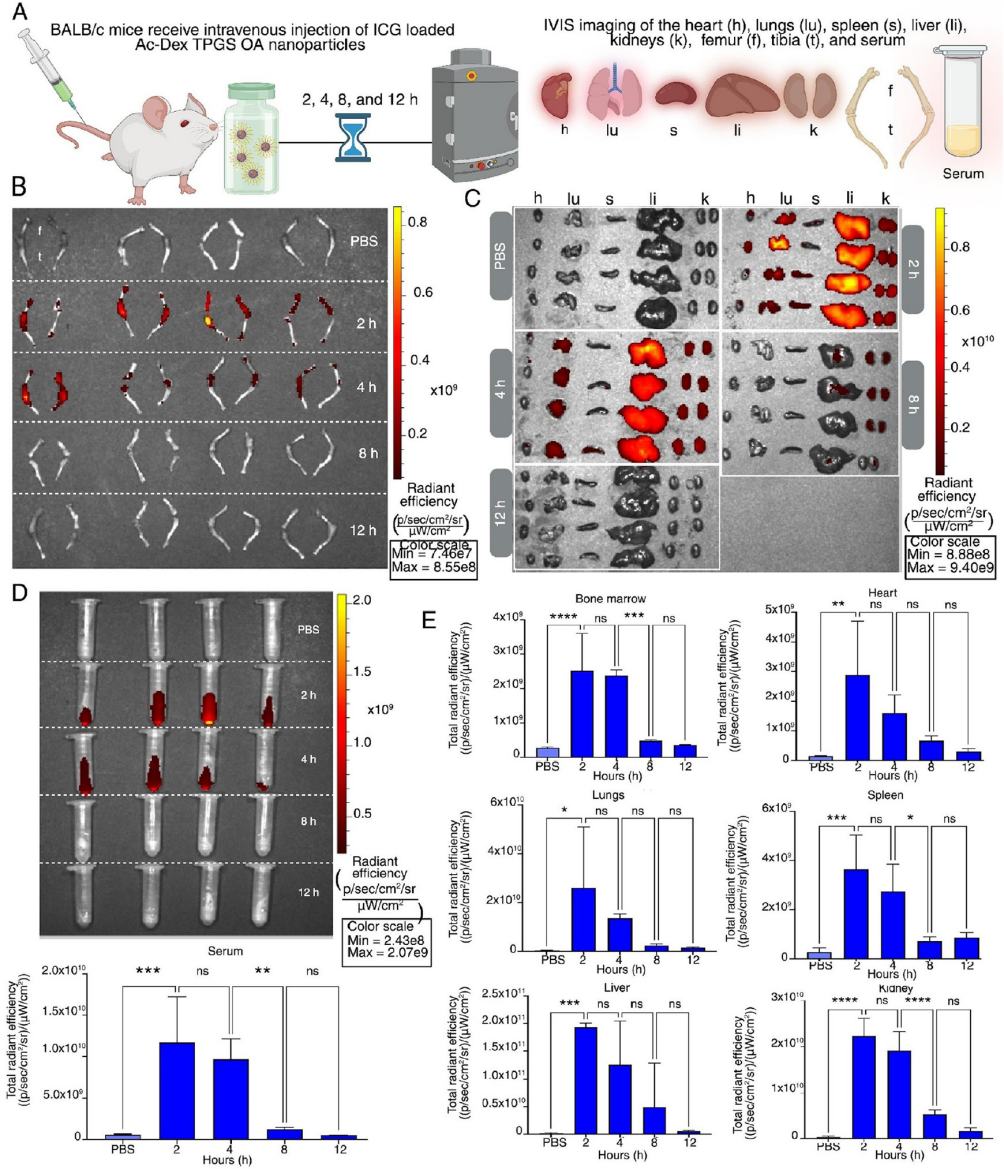
Time-dependent biodistribution of Ac-Dex TPGS OA nanoparticles. (A) Schematic of study design depicting intravenous administration of ICG-loaded Ac-Dex TPGS nanoparticles at various time points (*n* = 4 per group), followed by IVIS imaging. (B) IVIS imaging comparing hindlimb (femur and tibia) accumulation of nanoparticles. (C) IVIS imaging of the heart (h), lungs (lu), spleen (s), liver (li), and kidneys (k). (D) IVIS imaging and quantification of total radiant efficiency of the serum. (E) Quantification of total radiant efficiency for the bone marrow and individual organs. Statistical significance was determined by one way ANOVA with Tukey’s multiple comparison test (*p < 0.05, **p < 0.005, ***p < 0.0005, ****p < 0.00005, ns = not significant) with data reported as mean ± s.d.

Given that the intraperitoneal (IP) injection remains a widely studied route of administration for testing of chemotherapeutics in the discovery phase, we investigated the fate of Ac-Dex TPGS OA nanoparticles following IP injection. We tested this in immunocompetent BALB/c mice and immunocompromised NSG mice, which are commonly employed in human xenograft cancer models.^38^ Following IP administration of ICG-loaded Ac-Dex TPGS OA nanoparticles, both BALB/c and NSG mice showed significant accumulation of the ICG payload in the bone marrow compared to PBS controls 4 h postinjection (Figure S10). This suggests that the immune system does not substantially impact bone marrow accumulation of Ac-Dex TPGS OA nanoparticles. Interestingly, Ac-Dex TPGS OA ICG signal remained detectable in the lungs, liver, spleen, and hindlimb bones (femur and tibia) following 24 h of injection (Figure S11). This slower clearance as compared to IV administration is likely attributed to systemic absorption that occurs in the peritoneal cavity and lymphatic transport.^39,40^ Hematological malignancies, such as B-cell ALL, result in profound immunosuppression; however, nonmalignant conditions, such as essential thrombocytopenia, do not.^41,42^ The ability to achieve consistent bone marrow accumulation across distinct models enhances the clinical translatability of Ac-Dex TPGS OA nanoparticles, especially for the treatment of hematological diseases.

### 2.4 Evaluation of Biocompatibility Following 7 Day Administration of Intravenous Ac-Dex TPGS OA Nanoparticles

For successful clinical translation of novel delivery platforms, biocompatibility testing is highly relevant, especially for therapies that need repeated dosing and higher systemic exposure. To minimize variability in absorption and standardize systemic exposure, we chose intravenous administration as the dosing method. We assessed the biocompatibility by injecting mice daily with Ac-Dex TPGS OA nanoparticles, Ac-Dex TPGS nanoparticles, or PBS vehicle for 7 days. Blood chemistry tests were conducted 24 h after the last injection. Nephrotoxicity and hepatotoxicity were evaluated using serum creatinine (Figure 4A), blood urea nitrogen (Figure 4B), and alanine aminotransferase (Figure 4C). All three treatment groups remained below toxicity thresholds for each assay. These findings indicate that neither the polymeric component (Ac-Dex TPGS) nor the lipid surface modification (OA) affects systemic toxicity. To further confirm the biocompatibility of our formulations, we performed histopathological analyses of liver, lungs, spleen, and kidney tissue (Figure S12). Formalin-fixed and paraffin-embedded sections were stained with H&E and analyzed by a pathologist in a blinded manner to observe any pathological lesions and assess whether there were differences between the PBS, Ac-Dex TGPS and Ac-Dex TGPS OA groups. All the observed tissues demonstrated no gross or microscopic lesions. Furthermore, no significant differences were observed between the Ac-Dex TGPS and Ac-Dex TGPS OA groups. Biodistribution was examined after 7 days of ICG-loaded Ac-Dex TPGS OA nanoparticles or Ac-Dex TPGS nanoparticles administration; samples were collected 24 h after the last dose. The femur, tibia, heart, lungs, liver, spleen, and kidneys were collected and evaluated for fluorescent signal using IVIS imaging (Figure S13). Minimal or undetectable signal of ICG was observed in all the treatment groups, indicating no accumulation of the encapsulated payloads and appropriate systemic clearance following repeated dosing.

**Figure 4:**
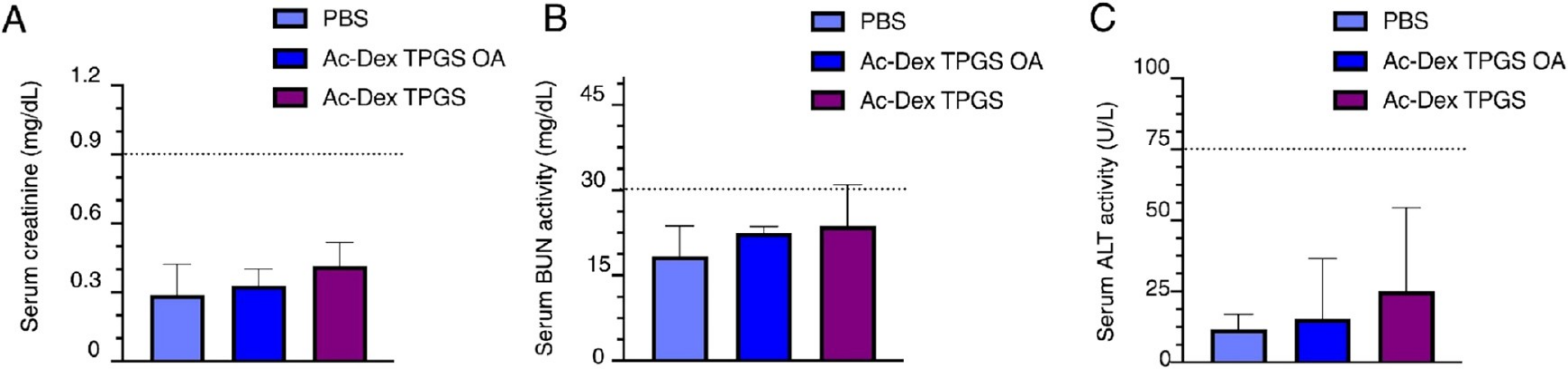
Evaluation of systemic biocompatibility following repeated exposure to Ac-Dex TPGS OA nanoparticles through intravenous administration. (A) Serum creatinine analysis (B) Serum blood urea nitrogen (BUN) analysis. (C) Alanine aminotransferase (ALT) analysis. Biochemical analysis treatments included Ac-Dex TPGS OA (*n* = 3), Ac-Dex TPGS (*n* = 3), and PBS (*n* = 3). The upper limit of normal physiological values is denoted by the dotted lines, indicating no nephrotoxicity or hepatotoxicity when these values are not exceeded.

### 2.5 Intracellular Release of Payloads Occurs in Acute Lymphoblastic Leukemia Cell Lines

In this study, we chose B-cell acute lymphoblastic leukemia (ALL) as a hematological malignancy model to validate the functional efficiency of Ac-Dex TPGS OA nanoparticles. As Ac-Dex TPGS OA nanoparticles were pH-responsive, we first evaluated *in vitro* intracellular release of encapsulated payload in two distinct B-cell ALL cell lines, REH and SUPB15.

We incubated rhodamine dye-loaded Ac-Dex TPGS OA nanoparticles with REH or SUPB15 cells for 24 h and examined the cytosolic release through confocal microscopy. Ac-Dex TPGS OA nanoparticles were taken up by both REH and SUPB15 cells (Figure 5A,B) and showed a cytosolic release, indicated by the diffused rhodamine signal inside cells. This cytosolic release can be attributed to the particle degradation that occurs in the acidic endolyosomal conditions.

**Figure 5:**
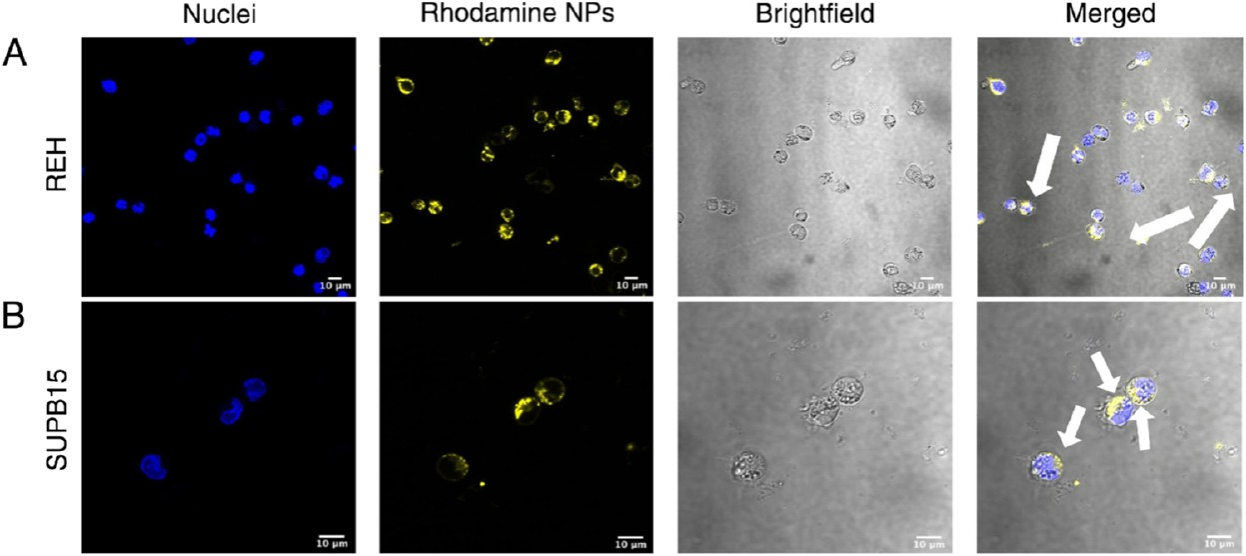
Cytosolic release by Ac-Dex TPGS OA nanoparticles loaded with rhodamine at 4 h. The nuclei are denoted in blue and rhodamine-loaded nanoparticles in yellow. The brightfield shows the cell morphology and cell membrane. The merged panel includes all channels in an overlay. The cytosolic release is highlighted by white arrows (A) Rhodamine-loaded nanoparticles release in REH cells. (B) Rhodamine-loaded nanoparticle release in SUPB15 cells. Live cell imaging was performed with the 40× objective, and the scale bar is 10 μm.

### 2.6 Biodistribution of Ac-Dex TPGS OA Nanoparticles in a Leukemic Mouse Model

The bone marrow microenvironment is inherently complex, and hematological malignancies such as B-cell ALL further change their vasculature and increase angiogenesis.^43^ Changes to the normal pathophysiology can greatly alter drug delivery; therefore, it is important to validate the distribution profile of these nanoparticles in the presence of leukemic burden. To test this, we have utilized a human xenograft B-cell ALL model where NSG mice were engrafted with human REH B-cell ALL cells. We injected NSG mice with 2.5 × 10^6^ REH cells and waited 21 days for the leukemic burden (Figure 6A) before administering ICG-loaded Ac-Dex TPGS OA nanoparticles via intravenous (IV) or intraperitoneal (IP) route. The route of administration was reevaluated for nanoparticle delivery to gain greater insight into the impact of the disease model.

**Figure 6:**
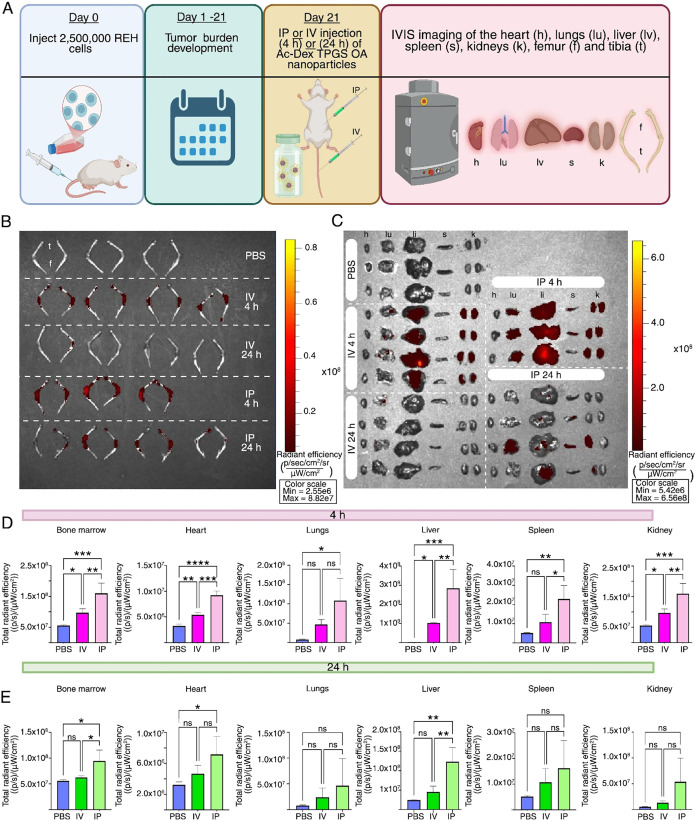
ICG-loaded Ac-Dex TPGS OA nanoparticle biodistribution in B-cell ALL mice. (A) Schematic of study design. NSG mice were injected with 2.5 × 10^6^ REH cells. Routes of administration were intraperitoneal (IP) or intravenous (IV) and included both 4 and 24 h sacrifice times. (B) IVIS imaging comparing hindlimb (femur and tibia) accumulation of nanoparticles. (C) IVIS imaging of the heart (h), lungs (lu), spleen (s), liver (li), and kidneys (k). (D) Quantification of total radiant efficiency 4 h post administration. (E) Quantification of total radiant efficiency 4 h post administration. Statistical significance was determined by one way ANOVA with Tukey’s multiple comparison test (*p < 0.05, **p < 0.005, ***p < 0.0005, ****p < 0.00005, ns = not significant) with data reported as mean ± s.d. *n* = 3–4 mice per group.

The hindlimbs (femur and tibia), heart, lungs, liver, spleen, and kidneys were collected at 4 and 24 h postinjection of nanoparticles, and the fluorescence was quantified through IVIS imaging (Figure 6B,C). After 4 h, the IP route demonstrated greater fluorescence signal in bone marrow, liver, spleen, and kidneys compared to the IV route (Figure 6D). Surprisingly, a significant retention of nanoparticles in the heart was observed following IP delivery; however, the overall signal remains relatively low as compared to other organs. Importantly, both IV and IP administration demonstrated significant accumulation in the bone marrow in this disease model. The fluorescent signal decreased significantly by 24 h in all the organs, indicating nanoparticle clearance. Interestingly, a significant fluorescent signal was observed after 24 h in the bone marrow, liver, and heart with IP administration as compared to IV administration (Figure 6E). These results suggest that the route of administration impacts organ-level biodistribution of nanoparticles in leukemic mice. Also, this data supports the suitability of IP and IV administration of Ac-Dex TPGS OA nanoparticles when therapeutic accumulation in the bone marrow is warranted. IP administration likely has a prolonged effect due to the slower release of nanoparticles from the peritoneal cavity, while IV experiences a more rapid clearance; these routes may be further explored based on the target therapeutic. Achieving significant passive accumulation in the bone marrow further highlights the clinical potential and translatability of Ac-Dex TPGS OA nanoparticles in hematological diseases.

### 2.7 Functional Outcomes and Therapeutic Efficacy of Vincristine Loaded Ac-Dex TPGS OA Nanoparticles in a Leukemia Model

Given the capability of Ac-Dex TPGS OA nanoparticles to accumulate in the bone marrow, we evaluated the functional efficacy in a human B-cell ALL xenograft model using the antileukemic drug, vincristine. Vincristine is currently used as a critical component in the standard of care regimen for all ages in treating B-cell ALL in clinics. However, vincristine suffers from a narrow therapeutic index and dose-dependent peripheral neurotoxicity, and therefore necessitates the development of novel vincristine formulations such as nanoparticles.^44^ The vincristine loaded Ac-Dex TPGS OA nanoparticles possess similar characteristics as unloaded nanoparticles with a size of 182 d.nm, surface neutral charge, low PDI, and vincristine encapsulation efficiency of 42.2% (Figure S14)

Human B-cell ALL engraftment was performed in NSG mice using intravenous injection of REH cells. After 1 week of engraftment, mice were intravenously administered treatments triweekly for 2 weeks with either PBS, blank Ac-Dex TPGS OA nanoparticles, free soluble vincristine, or vincristine loaded Ac-Dex TPGS OA nanoparticles (Vinc-NPs) (*n* = 10) (Figure 7A). It is important to note that vincristine is administered intravenously in clinics, and therefore, we have chosen the intravenous route of administration for treatment groups.

**Figure 7:**
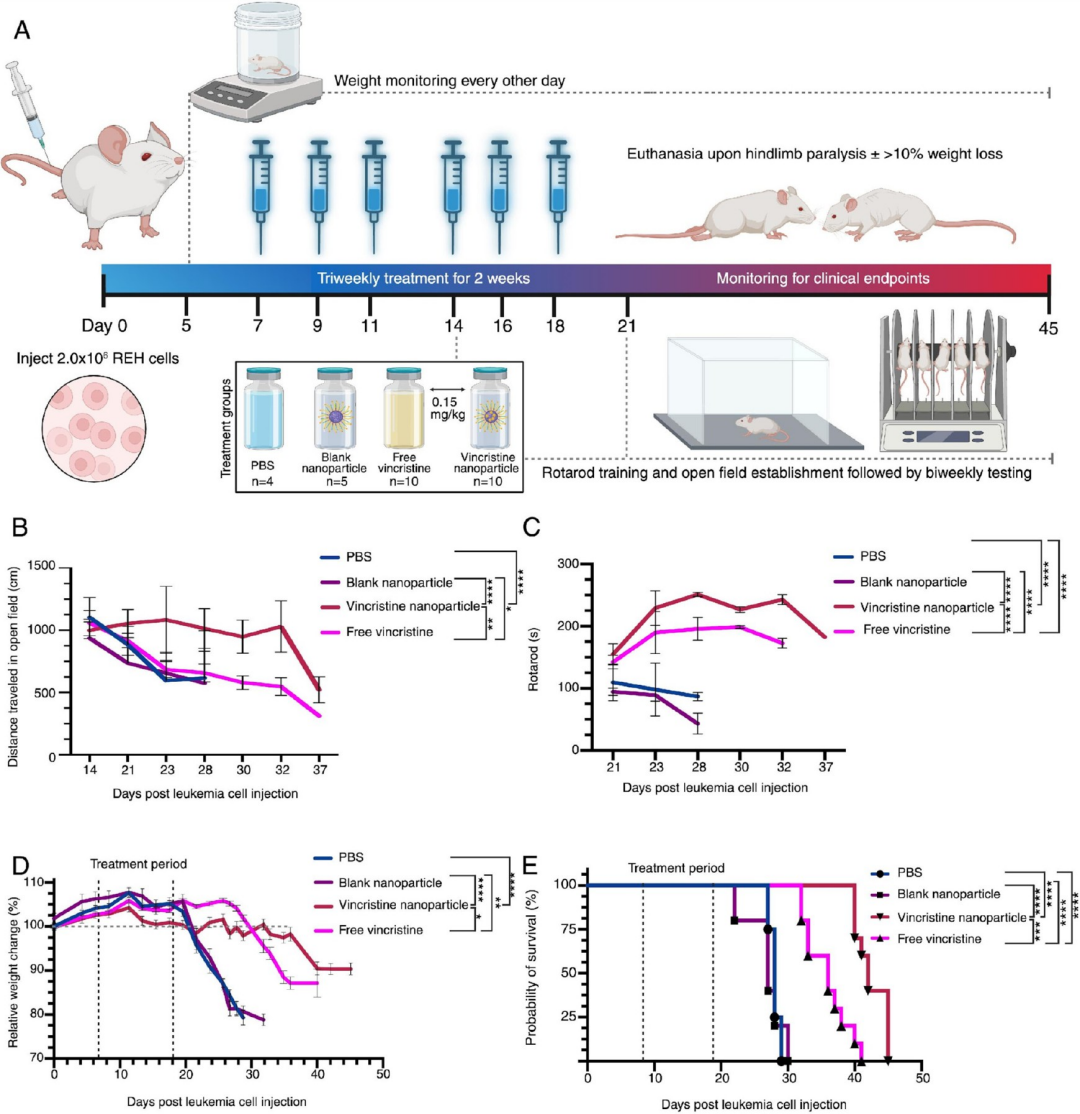
Functional outcome preservation and survival benefit of vincristine loaded Ac-Dex TPGS OA nanoparticles in a B-cell ALL model. (A) Schematic of study design. NSG mice were injected with 2.0 × 10^6^ REH cells. Control groups include PBS (*n* = 4) and blank nanoparticles (*n* = 5). The treatment groups are free vincristine (*n* = 10), and vincristine loaded into the Ac-Dex TPGS OA nanoparticle (*n* = 10). Vincristine dosing was 0.15 mg/kg and administered in six total doses over 2 weeks. (B) Open field testing assessing total distance traveled (cm) during 10 min sessions. (C) Locomotor coordination on rotarod measured by time to fall (s), with a 300-s cutoff. (D) The percentage change in relative body weight over time. Statistical significance was determined by two-way ANOVA with Tukey’s multiple comparison test. (E) Kaplan–Meier survival curve. Statistical significance was determined by log-rank test. Data are expressed as mean ± SEM (*p < 0.05; **p < 0.005; ***p < 0.0005; ****p < 0.0001).

Vincristine is often dosed *in vivo* within a range of 0.15 to 0.5 mg/kg.^45,46^ It has been observed that dosing at 0.15 mg/kg every other day or biweekly has improved tolerability compared to high doses given once weekly.^47,48^ Based on this, we utilized 0.15 mg/kg as the vincristine dose for every injection. Following treatments, behavioral assessments over time were performed on mice using open-field (Figure 7B) and rotarod performance (Figure 7C) tests. Additionally, weight change (Figure 7D) and survival (Figure 7E) were recorded. Euthanasia was performed upon the development of hindlimb paralysis or >10% body weight loss.

Open field testing and the rotarod are assays that can monitor locomotor impairment, which may arise due to increased leukemic burden or neurotoxicity associated with vincristine.^49–52^ Open field testing revealed a progressive decline in movement that coincided with advanced disease. The functional outcomes included the cumulative distance traveled (Figure 7B), speed (Figure S15), and total number of hindlimb rearings (Figure S16). Mice treated with Vinc-NPs retained significantly more mobility, as indicated by the cumulative distance traveled surpassing free vincristine and controls. This is especially evident starting around day 28 postleukemia cell injection and beyond when comparing Vinc-NPs with free vincristine (Figure S17). Rearing frequency retention was also significantly higher in the Vinc-NP group relative to other treatments. The average movement speed did not differ between groups, which may be due to the spontaneity involved in movement that has a high variability from averaging time points. Rotarod testing demonstrated that the Vinc-NP group outperformed all other treatments for locomotive ability. This suggests there may be delayed disease progression, reduction of peripheral neuropathy commonly associated with vincristine, or a combination of both. These data are promising for the potential to improve a patient’s quality of life. The sustained behavioral functionality observed in the Vinc-NPs may reflect the benefit of targeted drug delivery, resulting in more consistent local exposure of a therapeutic through passive accumulation. While not a locomotor functional outcome, alopecia is also commonly reported as an adverse effect of systemic vincristine administration.^53^ We observed that mice treated with the Vinc-NPs experienced less alopecia than the free vincristine group (Figure S18). These results suggest that localized drug delivery of vincristine may decrease the side effects associated with free vincristine biodistribution.

Body weight is one of the critical clinical predictive factors for prognosis and leukemic burden.^54,55^ The mice were weighed approximately every other day; their weights remained stable until disease onset, at which point they experienced a steady decline. Around day 23, the control groups (PBS and the blank nanoparticle) experienced extreme weight loss that coincided with advanced disease burden. All mice in the control group had to be euthanized by day 30, 1 week after this profound weight loss occurred, signaling aggressive disease progression when left untreated. The mice treated with free vincristine were able to maintain weight until around day 28, at which point their weight rapidly decreased. Interestingly, the Vinc-NPs demonstrated a stable weight and had significantly less weight loss at the time of euthanasia compared to PBS and cumulatively compared to all treatments (Figures S19 and 7D). To assess the survival, mice were monitored until the humane end points were reached and euthanasia occurred. The mice treated with PBS or the blank nanoparticle reached clinical end points significantly faster than both free vincristine and Vinc-NPs, as seen in Figure S20. Vinc-NPs significantly enhanced survival in leukemic mice with a median survival time of 46 days as compared to free vincristine with a median survival time of 31 days (Figure 7E). Interestingly, we did not see any significant difference in survival between PBS and blank nanoparticle groups.

The increase in survival and preservation of locomotive abilities showcases the delivery platform’s enhancement of vincristine’s clinical performance. This is especially relevant as the previous FDA-approved vincristine liposomal formulation, Marqibo, was pulled from the market in 2021 due to failure to verify therapeutic benefit.^56^ Vincristine is a cornerstone treatment in the Ph-negative B-cell ALL treatment algorithm for remission induction.^57^ Improving the efficacy and reducing adverse effects should be further explored for vincristine, given the current lack of approved advanced drug delivery formulations. Future studies are needed to explore the underlying mechanism of these functional outcomes, which may include nerve conduction testing or histopathological examination of structural damage. Additional studies should include other small molecules, as well as coencapsulation or coadministration of treatments.

## 3 Conclusion

This work showcases the integration of OA, an endogenous bone marrow fatty acid, into polymeric Ac-Dex nanoparticles, enabling passive, yet selective, accumulation of the encapsulated payload in the bone marrow of both immunocompetent and immunocompromised mice, but most notably, in the presence of an established leukemic burden. Ac-Dex TPGS OA nanoparticles are stable, inherently nontoxic, and possess excellent biocompatible and biodegradable properties, allowing multiple systemic administrations *in vivo*. In a B-cell ALL model, vincristine loaded Ac-Dex TPGS OA nanoparticles improved overall survival, preserved locomotor function, and reduced terminal weight loss compared to free systemic vincristine. Overall, this platform holds a great translational potential in treating bone marrow localized diseases, and future studies warrant detailed characterization of the encapsulation of diverse clinically relevant therapeutics and their functional outcomes.

## 4 Experimental Section

### 4.1 Materials

Dextran from *Leuconostoc mesenteroides* (MW 9000–11,000), 2-methoxypropene, d-α-Tocopherol poly­(ethylene glycol) 1000 succinate, rhodamine B, and d-mannitol 97+%, Free Fatty Acid Assay Kit, Serum Creatinine Colorimetric Detection Kit, Urea Nitrogen (BUN) Colorimetric Detection Kit, Alanine Aminotransferase (ALT/GPT) Activity Kit were purchased through Sigma-Aldrich (Burlington, MA). DiD perchlorate was obtained from MedChemExpress (Monmouth Junction, NJ). d-(+)-trehalose dihydrate, and d-(+)-Sucrose, were purchased through TCI America (Portland, OR). Palmitic acid, oleic acid, DSPE-mPEG, and Vincristine (Sulfate) were purchased from Cayman Chemical (Ann Arbor, Michigan). The confined impingement jet mixer was obtained from Holland Applied Technologies (Burr Ridge, IL). Indocyanine green was purchased from Tocris Bioscience (Minneapolis, Minnesota). The Zombie Aqua and Zombie Red stain fixable viability kit was obtained from BioLegend (San Diego, California). NucBlue Live ReadyProbes Reagent was purchased through Thermo Fisher Scientific (Waltham,MA). PE/Cy5 CD3 antibody, PE/Cy7 CD4 antibody, Pacific Blue CD45 antibody, Alexa Fluor 488 Ly-6C antibody, and CD16/32 antibodies were obtained from BioLegend (San Diego, California). Novafluor Blue CD19, PerCp-eFluorTM 710 Ly-6G, Super BrightTM 436 CD11c, Super BrightTM 600 F4/80, Super BrightTM 702 CD11b and Super BrightTM CD8a antibodies were obtained from ThermoFisher Scientific (Waltham, MA). Pacific Blue CD45, PerCP-eFluor 710, Alexa Fluor 488 Ly6, PE CD19, SuperBright 436 CD11, and SuperBright 600 F4/80 were obtained from BioLegend (San Diego, California). PE-CF594 CD19, APC-R70 CD45, FITC CD22 were obtained through BD Biosciences (Franklin Lakes, NJ).

### 4.2 Methods

#### 4.2.1 Acetalated Dextran Synthesis

Dextran (*M*_w_ 10,500 g/mol, 1.00 g, 0.095 mmol) is dissolved in anhydrous DMSO (10 mL) under nitrogen gas, along with pyridinium *p*-toluenesulfonate (15.6 mg, 0.062 mmol) and 2-methoxypropene (3.4 mL, 37 mmol).^58,13^ The solution was stirred for 30 min and then quenched using triethylamine (1 mL, 7 mmol). The solution was then precipitated in distilled water (150 mL). The precipitate was centrifuged at 1000*g* for 10 min, and the pellet was washed twice with water (pH 9) before lyophilization to remove residual water and collect the Ac-Dex. The reaction yield was >90% and ^1^H NMR using deuterium chloride (DCl) and deuterium oxide (D2O) as solvents determined the cyclic acetal coverage to be 57.3%.

#### 4.2.2 Formulation of Ac-Dex OA Nanoparticles

Ac-Dex, TPGS, and OA were used to form Ac-Dex OA nanoparticles, and using the FNP technique.^13^ The organic phase was formulated using 500 μL of ethanol. The aqueous phase was formulated with PBS. Ac-Dex or OA was added to the organic phase, while TPGS was added to the aqueous phase. The organic and aqueous phases were impinged in a confined impingement mixer and the nanoparticles were spun overnight to evaporate the organic solvent. The nanoparticles were then centrifuged and washed twice with deionized water and resuspended in PBS to a final volume of 2 mL before characterization. After formulation, nanoparticles were stored at 4 °C in PBS; if lyophilized, the formulation was stored at −20 °C. Cryoprotectant optimization included trehalose, mannitol, and sucrose at 5–20% w/v. The samples were transferred to a 10 mL vial and placed at −80 °C for 1 h. Once the samples were frozen, they were moved to the Lyovapor L-200 lyophilizer and left overnight. For serum stability, 100 μL of the nanoparticles loaded with DiD were added to 2 mL microcentrifuge tubes with 250 μL of 10% fetal bovine serum (FBS). The microcentrifuge tubes were placed on an orbital incubator shaker (Benchmark Scientific Incu-Shaker Mini) for various time points (*n* = 3 per condition) at 37 °C. After incubation, the microcentrifuge tubes were centrifuged at 5000*g* for 5 min. 50 μL of the supernatant was collected into 96-well clear-bottom polystyrene plates (*n* = 3 per sample). The fluorescence for each sample was measured using a SpectraMax iD5 microplate reader.

#### 4.2.3 Physicochemical Characterization

Dynamic light scattering and electrophoretic light scattering were used to measure the size and zeta potential using the Malvern Zetasizer Nano ZS. For size and polydispersity index (PDI), 20 μL nanoparticle samples were added to 1 mL of PBS. A capillary cuvette was used to measure the zeta potential. All readings were taken in triplicate. Encapsulation efficiency was determined by comparing the fluorescence before and after the samples were processed through centrifugation. All fluorescence readings were taken using an iD5Max microplate reader. DiD perchlorate (λEx = 644 nm; λEm = 665 nm), 0.4 mg, was added to the organic phase. Rhodamine 6B (λEx = 546 nm; λEm = 568 nm), 1 mg, was added to the aqueous phase. Indocyanine green (λEx = 789 nm; λEm = 813 nm), 1 mg, was added to the aqueous phase. The encapsulation efficiency of vincristine sulfate (λ excitation = 295 nm) was determined by UV absorbance using a NanoDrop One Spectrophotometer (Thermo Fisher Scientific). One mg of Vincristine was added to the aqueous phase of Ac-Dex TPGS OA nanoparticles. After formulation and processing through centrifugation to remove unencapsulated drug, a standard curve of vincristine was used to determine the concentration (*R* ^2^ > 0.99). For fluorophore retention, Ac-Dex nanoparticles were loaded with DiD or Rhodamine and analyzed before lyophilization. From a 2 mL of the initial formulation, 1 mL was stored overnight at 4 °C, and the other 1 mL of the formulation was lyophilized with 10% (w/v) mannitol. The lyophilized cake was rehydrated to form nanoparticles and was centrifuged to remove the free drug, and the pellet was resuspended in PBS and analyzed for DiD or rhodamine retention. Transmission electron microscopy samples were applied to UV-treated grids (Ted Pella, 01840-F) and stained immediately with 1% aqueous uranyl acetate, blotted, and air-dried. Images were acquired using a JEOL JEM-1010 Transmission Electron Microscope.

#### 4.2.4 Oleic Acid Retention

Oleic acid was quantified using a free fatty acid colorimetric assay, as per the manufacturer’s protocol. Oleic acid (%) was calculated as (measured OA in the final fformulation/added OA to initial formulation) × 100.

#### 4.2.5 pH-Responsive Drug Release Studies

200 μL of nanoparticles were added to 2 mL microcentrifuge tubes with 500 μL of the designated pH buffer. Each acidic pH buffer was prepared by adding hydrochloric acid to PBS, while basic pH buffers were made by adding sodium hydroxide to PBS. The microcentrifuge tubes were placed on an orbital incubator shaker (Benchmark Scientific Incu-Shaker Mini) for various time points (*n* = 3 per buffer condition) at 37 °C. After incubation, the microcentrifuge tubes were centrifuged at 5000*g* for 5 min. 50 μL of the supernatant was collected into 96-well clear-bottom polystyrene plates (*n* = 3 per sample). The fluorescence for each sample was measured using an iD5Max microplate reader.

#### 4.2.6 Cell Culture and *In Vitro* Imaging

REH-CRL-8286 B-cell acute lymphoblastic leukemia cells, as well as SUPB15 B-cell acute lymphoblastic leukemia cells (Ph+) were obtained from ATCC and were cultured with Roswell Park Memorial Institute 1640 (RPMI) supplemented with 10% fetal bovine serum (FBS) and 1% penicillin/streptomycin antibiotics. Cells were plated in either T25 or T75 nontreated tissue culture flasks. The cells were fed with additional media every 3 days. All cells were cultured at 37 °C, 5% CO_2_. The cytosolic release was analyzed using a Nikon A1R confocal fluorescence microscope (40× lens) equipped with three laser lines: 405 nm (NucBlue LiveReady Probe) (Invitrogen), 640 nm (DiD dye), and 550 nm (Rhodamine B). The brightfield (transmitted light) was also imaged. Galvano scanning, with a 1.2 pinhole, at 1/eighth frame/sec and a 512 size, was used during imaging. REH-CRL-8286 and SUPB15 cells were seeded at a density of 5 × 10^4^ cells per well, using 12-well nontreated tissue culture plates. Twenty μL of the nanoparticles was added to each well, and the plates were left overnight to incubate at 37 °C, 5.0% CO_2_. Following incubation, the wells were aspirated, and the cell suspension was centrifuged at 800 rpm for 5 min. The supernatant of dead cells and nonendocytosed nanoparticles was discarded, and the cell pellet was resuspended in Roswell Park Memorial Institute (RPMI) 1640. 300 μL of poly- d -lysine (PDL) (Gibco) was added to each of the 8-well chambered slides and left out to incubate at room temperature for 1 h. The PDL was then aspirated, and the wells were washed three times with PBS. After the last wash, the remaining PBS was aspirated, and the coated chamber slide was left to dry overnight. When the coated plates were not immediately used, they were tightly wrapped in Parafilm. The cells adhered overnight and were incubated at 37 °C, 5.0% CO_2_. The media was then aspirated, and the wells were washed once with PBS. One drop of NucBlue per well was added directly before imaging. Confocal images were subsequently processed using the ImageJ software (Version 2.1.0/1.53c).

#### 4.2.7 *In Vivo* Studies

All animal studies were approved by the West Virginia University Institutional Animal Care and Use Committee (IACUC) and carried out under relevant institutional guidelines, regulations, and approved protocols. Six to twelve weeks old male NOD.Cg-*Prkdc* ^*scid*^ *Il2rg* ^*tm1Wjl*^/SzJ (NSG) mice were used in leukemic burden and biodistribution studies. Clinical end points for leukemia burden studies included the development of hindlimb paralysis or >10% loss of body weight. Six to twelve weeks old male BALB/c mice were used in traditional biodistribution studies. Euthanasia for all animals occurred via cervical dislocation or through terminal cardiac puncture when blood collection was warranted for serum collection.

#### 4.2.8 IVIS/CT Imaging

Mice were intravenously injected through the lateral tail vein or intraperitoneally either with 100 μL of indocyanine green (ICG)-loaded nanoparticles or 100 μL PBS control. For whole body imaging, mice were anesthetized using 2.5% Isoflurane. The hindlimbs (femur and tibia), liver, kidney, lungs, heart, and spleen were harvested for *ex vivo* IVIS imaging using the IVIS SpectrumCT (PerkinElmer). The total radiant efficiency ((p/s)/(μW/cm^2^)) was reported to measure biodistribution through region of interest (ROI) quantification through Living Image v4.7.4.

#### 4.2.9 Serum Analysis And Histology

Serum, to test for liver and kidney damage, was collected on day 8 (7 days of dosing blank nanoparticles +24 h). Blood was collected via terminal cardiac puncture on heavily sedated (isoflurane) mice. Whole blood was collected into a serum clotting collection tube and centrifuged at 10,000*g* for 5 min. Serum was stored at −20 °C until analysis. Serum creatinine, blood urea nitrogen, and alanine aminotransferase colorimetric assay kits were used following the manufacturer’s protocol. The organs were collected and fixed in 10% formalin before being embedded with paraffin. The organ tissue was sliced, mounted, and stained on slides by the WVU Pathology core.

#### 4.2.10 Bone Marrow Single Cell Suspension and Flow Cytometry

Mice were intraperitoneally injected with 100 μL DiD dye-loaded nanoparticles or PBS as a control. The hindlimbs (femur and tibia) were harvested from the mice and manually cleaned. The ends of each bone were cut with a scalpel. After excision, the bones were stored in 1 mL of complete Roswell Park Memorial Institute (RPMI) 1640 media in 5 mL centrifugation tubes. The bone was suspended via forceps over a 15 mL tube containing 5 mL of fresh RPMI media. A 27-gauge needle was carefully inserted into one end of the bone, and an additional 2.5 mL of fresh media was flushed through each bone cavity. The collected cells were spun down at 1000*g* for 5 min at 4 °C. The resulting supernatant was discarded, and the pellet was resuspended to the desired concentration using sterile PBS. The single-cell suspension was stained for various types of immune cell markers. All samples were measured using the Cytek Aurora instrument, and the data were analyzed using Cytobank community software v10.7.

#### 4.2.11 Malignant Biodistribution

Eight-week-old NSG female mice were injected intravenously with REH cells and were allowed to develop disease burden for 21 days. Mice were then injected intravenously or intraperitoneally with ICG-labeled nanoparticles and sacrificed 4 or 24 h postinjection. The hindlimbs (femur and tibia), heart, lungs, liver, spleen, and kidneys were harvested and imaged using IVIS Spectrum CT. Euthanasia was performed upon the development of hindlimb paralysis or >10% body weight loss.

#### 4.2.13 Survival Analysis

NSG mice were injected intravenously with REH cells, with treatment starting on day 7 postinoculation. The mice were treated triweekly for 2 weeks with either PBS, blank nanoparticles, free vincristine, or vincristine loaded nanoparticles. Vincristine was dosed at 0.15 mg/kg. Mice were monitored daily, and euthanasia was performed upon the development of hindlimb paralysis or >10% body weight loss.

#### 4.2.14 Rodent Behavior Assessment

NSG mice were transported to the behavioral core facilities and acclimated for 15 min before each testing session. Additional ABSL-2 cleaning and disinfection protocols were followed after each session. For the open field testing, mice were individually added to the center of a 16 × 16 × 15 in. polypropylene photo beam activity system (PAS; San Diego Instrument) chamber where they were free to ambulate for 10 min sessions. Horizontal and vertical movements were measured by determining the number of photobeams (16 photobeans in each axis) disrupted from two sets of infrared sensors on either side of the chamber. The chambers were located within closed boxes to control ambient noise and light. Total distance traveled, number of rearings, and XY speed were recorded for each animal. Each chamber was cleaned between subjects. Data was analyzed using PAS-open field software. Rotarod testing utilized a starting baseline rotational speed of 4 rpm and accelerated to a maximum rotational speed of 40 rpm over the course of 300 s. The mice were trained on the rotarod on day 14 postleukemia cell injection, with recorded measurements beginning on day 21 postleukemia cell injection. Testing was performed in triplicate for each time point. Mice were given a 15 min rest interval between each round of testing. Data are reported as latency to fall off the rod, recorded in seconds. While being tested, only mice from the same cage were on the rotarod at any given time. The rotarod was cleaned between subjects.

#### 4.2.15 Statistical Analysis

Statistical analyses were performed using GraphPad Prism software (version 10.4.1). Data are presented as mean ± s.d. unless otherwise stated. Statistical tests performed here and in the supplemental section were selected based on sample size, distribution, and variance. One-way ANOVA was utilized, followed by Post Hoc Tukey’s or šídák’s multiple comparison test, Brown-Forsythe one-way ANOVA with Dunnett’s multiple comparisons test, two-way ANOVA followed by Tukey’s or šídák’s multiple comparison test, ordinary unpaired *t*-tests, Kruskal–Wallis test with an uncorrected Dunn’s test, and Log-rank tests were also utilized for survival studies. Significance was determined with a *p*-value <0.05.

## Supplementary Material



## Notes

Particle optimization, oleic acid retention, TEM, cryoprotectant optimization, pH release, FBS release, size distribution, encapsulation efficiency, immune panel cellular uptake, biodistribution, IVIS, model development, open field testing speed, open field testing rearings, open field testing tracking maps, alopecia visualization, therapeutic efficacy weight change, days survival. (PDF)
