# Supplementary material for: Oleic Acid Integrated Acetalated Dextran Nanoparticles for Enhanced Chemotherapeutic Delivery to the Bone Marrow
Source: ACS Appl Mater Interfaces. 2025 Oct 14;17(45):61799–813. doi: 10.1021/acsami.5c16936 (PMC12616596; doi:10.1021/acsami.5c16936)
Supplement: Supplementary file 1 [file am5c16936_si_001.pdf]

Supporting Information

Oleic Acid Integrated Acetalated Dextran Nanoparticles for Enhanced Chemotherapeutic Delivery to the Bone Marrow

Krystal A. Hughes[a], William H. Pentz[a,b], Bishal Misra[a], Morgan Surface[c], Werner J. Geldenhuys[a,d], Salik Hussain[e,f], Sharan Bobbala[a] Email: sharan.bobbala@hsc.wvu.edu*

a Department of Pharmaceutical Sciences, West Virginia University School of Pharmacy, Morgantown, WV 26505, United States.

b School of Medicine, West Virginia University, Morgantown, West Virginia, 26506, USA

c Department of Clinical Pharmacy, West Virginia University School of Pharmacy, Morgantown, WV 26505, United States

d Department of Neuroscience, West Virginia University School of Medicine, Morgantown, WV 26505, United States.

e Department of Microbiology, Immunology and Cell Biology, West Virginia University School of Medicine, Morgantown, WV 26505, United States.

f Department of Physiology, Pharmacology and Toxicology, West Virginia University, Morgantown, WV 26505, United States

Table S1. The quantity of polymer, nonionic PEGylated surfactant, and lipid used during formulation optimization. Size (d.nm) and polydispersity index (PDI) were determined through dynamic light scattering. Size is reported as mean ± s.d. (*n*=3).

Ac-Dextran (mg)	TPGS (mg)	OA (mg)	Size (d.nm)	PDI
20	4	1	512 ± 76.7	0.488
20	1	4	10900 ± 6640	1.48
20	5	5	3060 ± 91.2	1.33
20	2	3	1320 ± 400	1.32
20	3	2	1030 ± 159	1.08
20	2.5	2.5	145 ± 57	0.15

Table S2. The quantity of polymer, nonionic PEGylated surfactant, and lipid used during formulation optimization when determining the impact of surfactant presence. Size (d.nm) and polydispersity index (PDI) were determined through dynamic light scattering. Size is reported as mean ± s.d (*n*=3).

Ac-Dextran (mg)	TPGS (mg)	OA (mg)	Size (d.nm)	PDI
20	-	5	4239 ± 1650	1.8
20	2.5	2.5	145 ± 57	0.15
20	5	-	264 ± 53	0.04

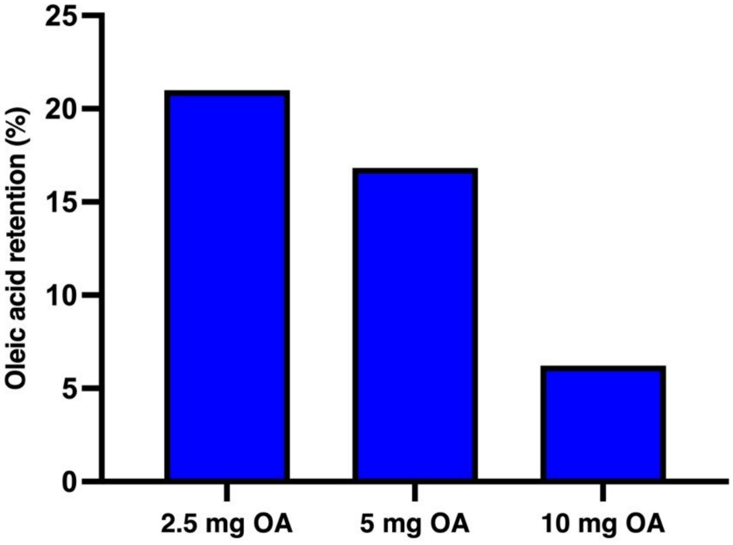

Figure S1 Oleic acid retention quantified through the percent of total OA retained after initial formulation.

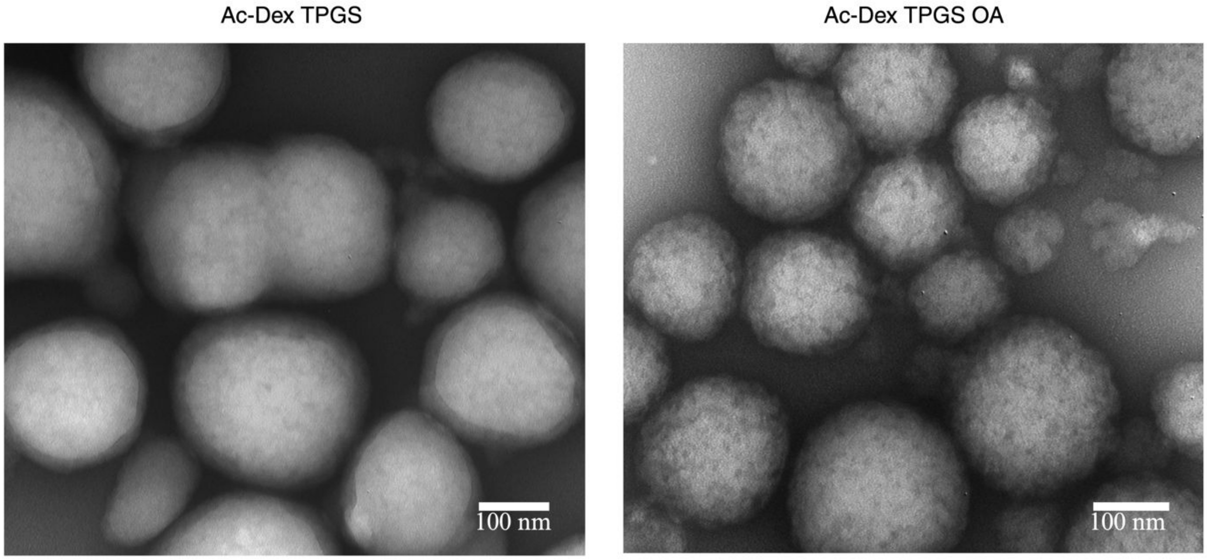

Figure S2. Transmission electron microscopy (TEM) images of Ac-Dex TPGS and Ac-Dex TPGS OA nanoparticles (Scale bar = 100 nm).

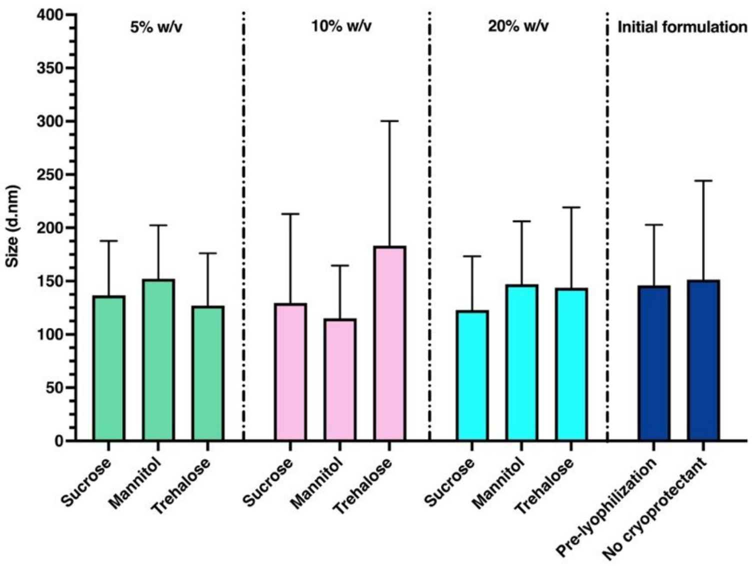

Figure S3. Cryoprotectant optimization compared to the initial formulation before lyophilization and after without cryoprotection. Size (d.nm) determined by DLS. Size is reported as mean ± s.d. (n=3).

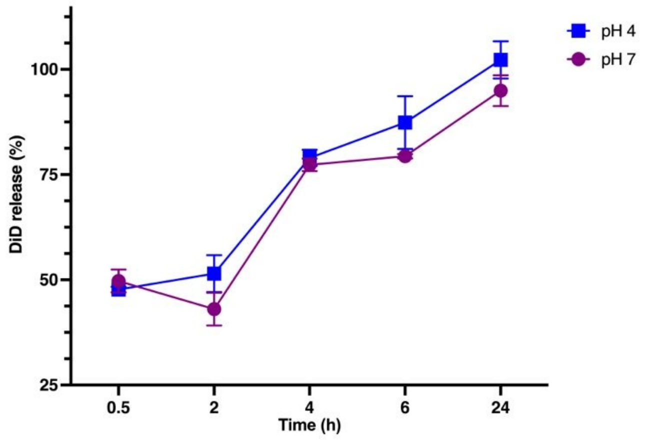

Figure S4. pH-responsive release profile of DiD from Ac-Dex TPGS OA nanoparticles. Nanoparticles were incubated in either a physiological buffer (pH 7.4) made from phosphate-buffered saline or an acidic buffer (pH 4.0) to simulate extracellular or intracellular environments. DiD release was quantified and compared in the two buffers at 0.5, 2, 4, 6, and 24 h and is presented as total release (%). The release is represented by mean ± s.d. (n = 3). Statistical significance was determined by unpaired t-test. There was no statistical significance at any time point.

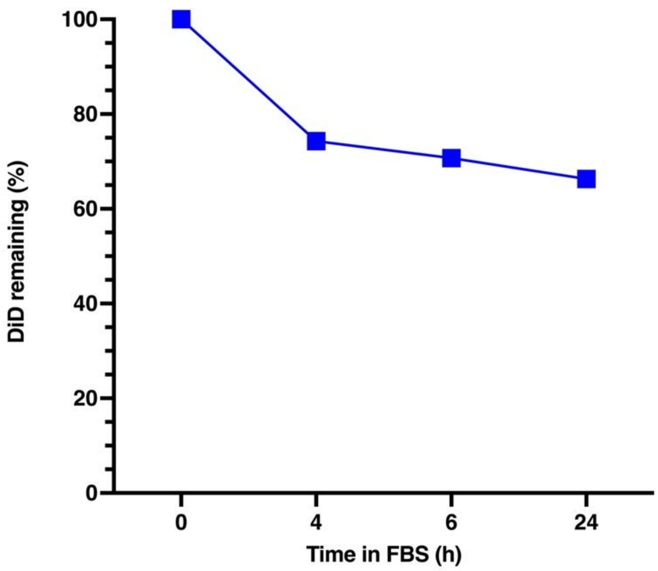

Figure S5. Release profile of DiD from Ac-Dex TPGS OA nanoparticles under a protein rich environment. Nanoparticles were incubated in 10% FBS solution with PBS. DiD release was quantified at 0.5, 2, 4, 6, and 24 h and is presented as DiD remaining (%).

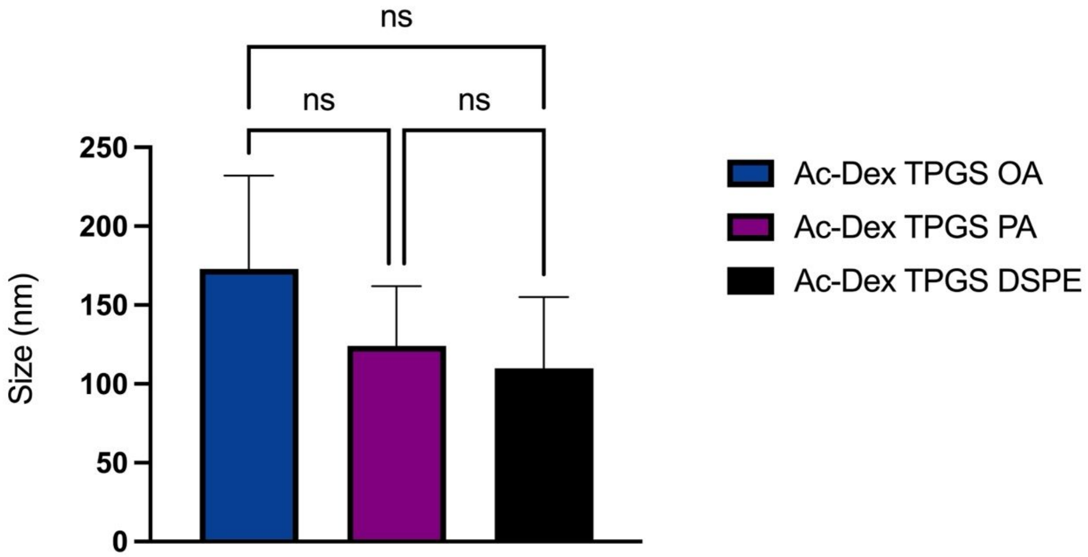

Figure S6 Size characterization of Ac-Dex TPGS nanoparticles with distinct lipid components. Nanoparticle tracking analysis of Ac-Dex TPGS formulated with oleic acid (OA), palmitic acid (PA), or DSPE-mPEG. Formulations ranged from 110 nm to 173 nm. Statistical significance was determined by one-way ANOVA. There was no statistical significance between formulations.

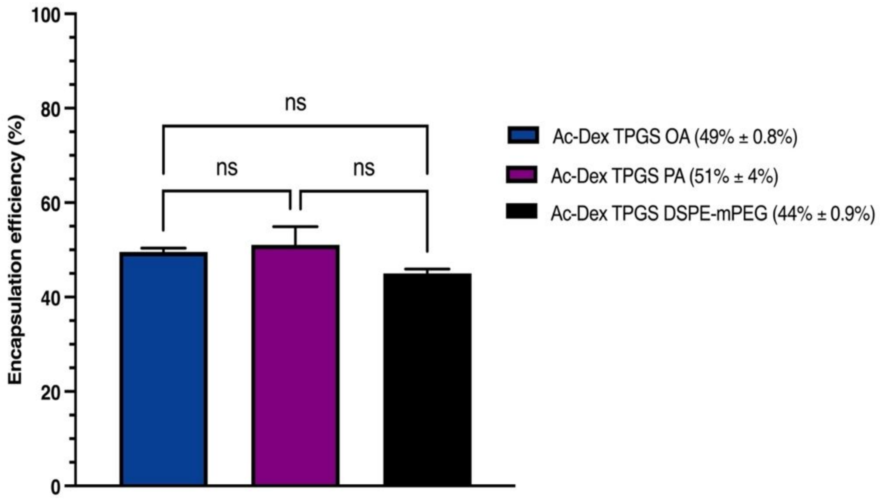

Figure S7 Encapsulation efficiency of ICG in Ac-Dex TPGS nanoparticles formulated with oleic acid (OA), palmitic acid (PA), and DSPE-mPEG. There was no statistical difference between formulations. Statistical significance was determined by a one way ANOVA with šídák’s multiple comparison test (ns = not significant) with data reported as mean ± s.d.

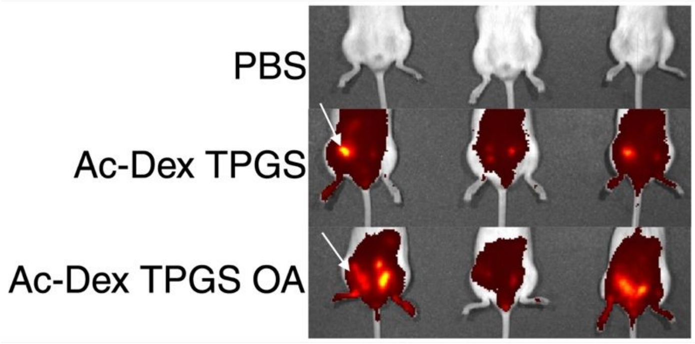

Figure S8. The presence of oleic acid increases the selective accumulation of nanoparticles in the hindlimbs. BALB/c mice were injected intraperitoneally with ICG-loaded Ac-Dex TPGS nanoparticles formulated with (*n* = 3) and without oleic acid (OA) (*n* = 3). Mice were imaged with whole body IVIS four hours post-injection. Ac-Dex TPGS OA exhibited a stronger localized distribution compared to Ac-Dex TPGS and a PBS control.

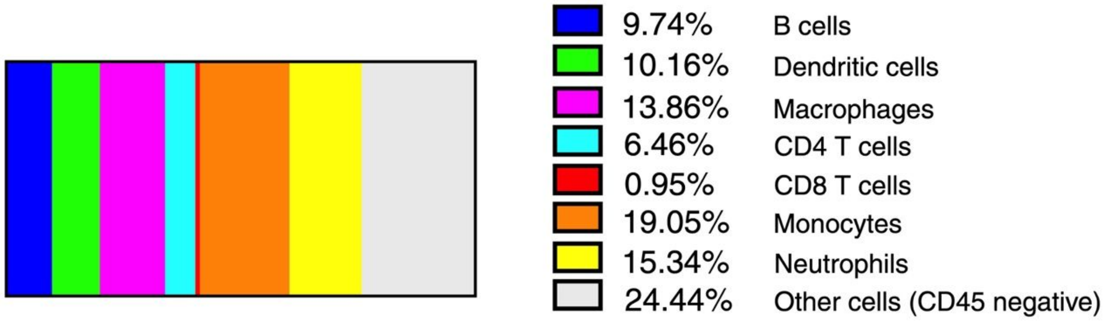

Figure S9 *In vivo* cellular uptake of DiD-loaded Ac-Dex TPGS OA nanoparticles in BALB/c mice (n = 2). Distribution of immune and non-immune cell populations in the bone marrow of healthy mice as determined by flow cytometry. Data reported as mean ± s.d.

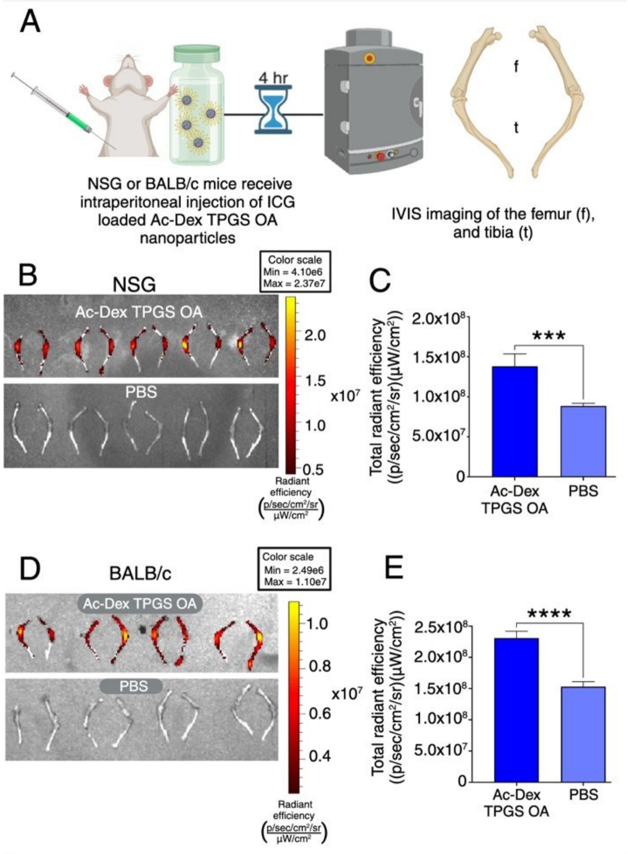

Figure S10 Bone marrow accumulation of ICG-loaded Ac-Dex TPGS OA nanoparticles occurs in immunocompetent and immunocompromised models. (A) Schematic of study design depicting intraperitoneal injections of ICG-loaded Ac-Dex TPGS nanoparticles and PBS, hindlimb bones (femur and tibia), were collected 4 hours after injection and analyzed with IVIS imaging. (B) IVIS imaging comparing hindlimb (femur and tibia) accumulation between Ac-Dex TPGS OA (n = 4) and PBS (n = 4) groups in NSG mice. (C) Quantification of total radiant efficiency for the bone marrow in NSG mice. (D) IVIS imaging comparing hindlimb (femur and tibia) accumulation between Ac-Dex TPGS OA (n = 4) and PBS (n = 4) groups in BALB/c mice. (E) Quantification of total radiant efficiency for the bone marrow in BALB/c mice. Statistical significance was determined by unpaired t-test (*** p<0.0005, *** p<0.00005) with data reported as mean ± s.d.

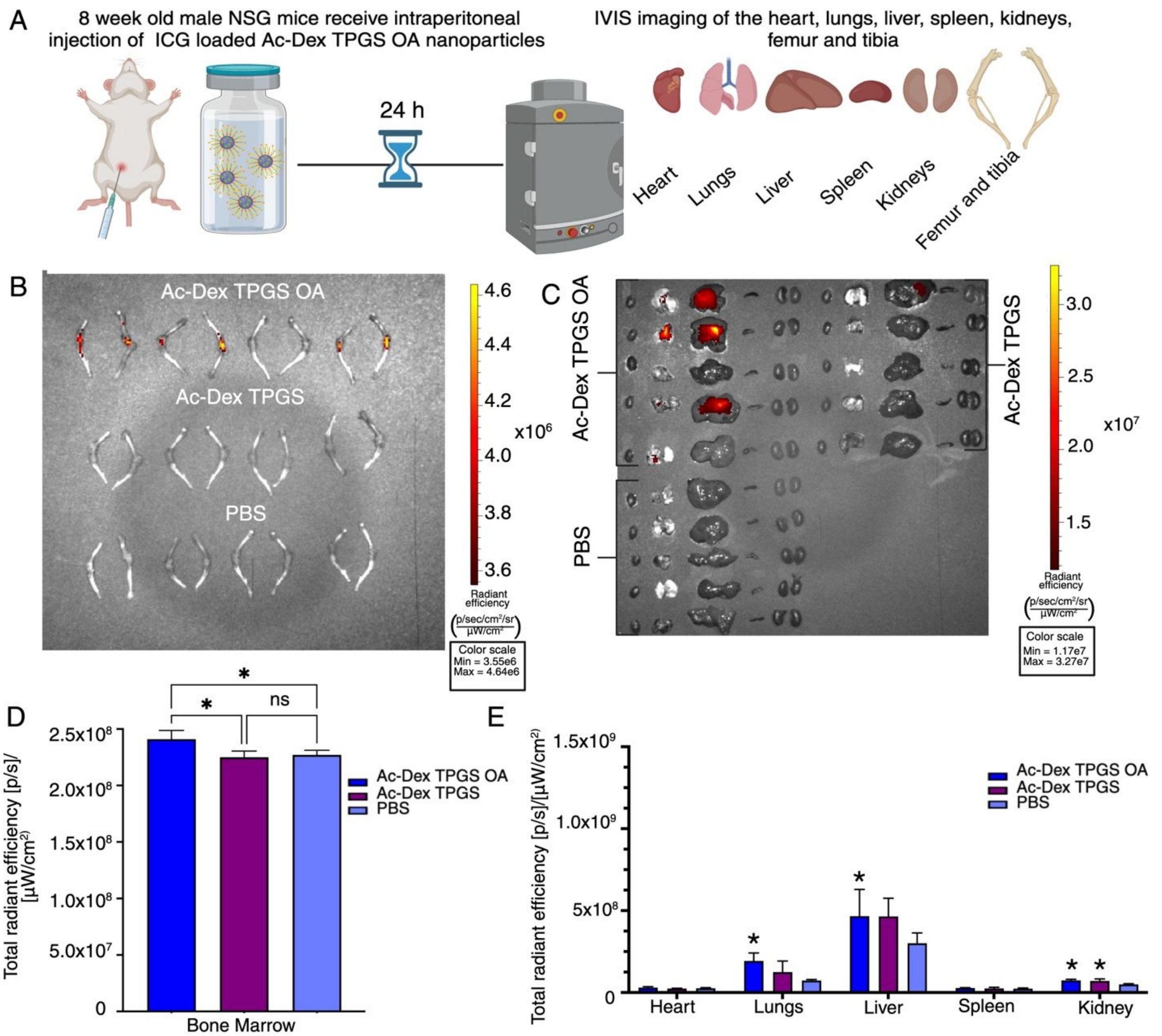

Figure S11 Biodistribution of Ac-Dex TPGS OA and Ac-Dex TPGS following intraperitoneal injection. (A) Schematic of study design depicting intraperitoneal injections of ICG-loaded Ac-Dex TPGS nanoparticles (*n* = 4), ICG-loaded Ac-Dex TPGS (*n* = 4), or PBS (*n* = 4). Hindlimb bones (femur and tibia), heart, lungs, liver, spleen, and kidneys were collected 24 hours after injection and analyzed with IVIS imaging. (B) IVIS imaging comparing hindlimb (femur and tibia) accumulation between treatment groups. (C) IVIS imaging of major organs. (D) Quantification of total radiant efficiency for the bone marrow and (E) organs. Statistical significance was determined by one way ANOVA with Tukey's multiple comparison test (* $p<0.05$) with data reported as mean ± s.d.

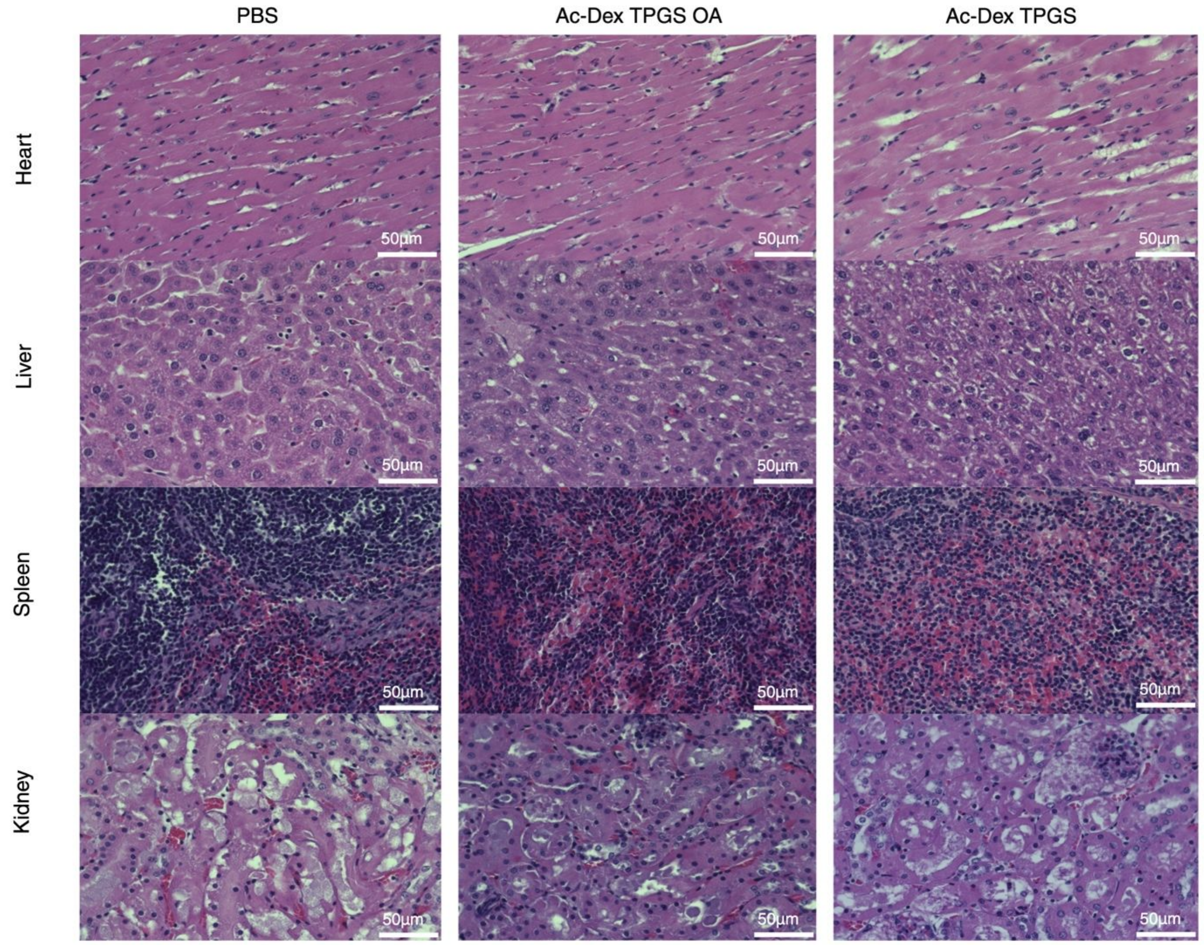

Figure S12 Evaluation of systemic biocompatibility following repeated exposure to Ac-Dex TPGS OA through intravenous administration. Representative images of the hematoxylin and eosin (H&E) stained sections of the heart, liver, spleen, and kidneys. Scale bar is 50 μm.

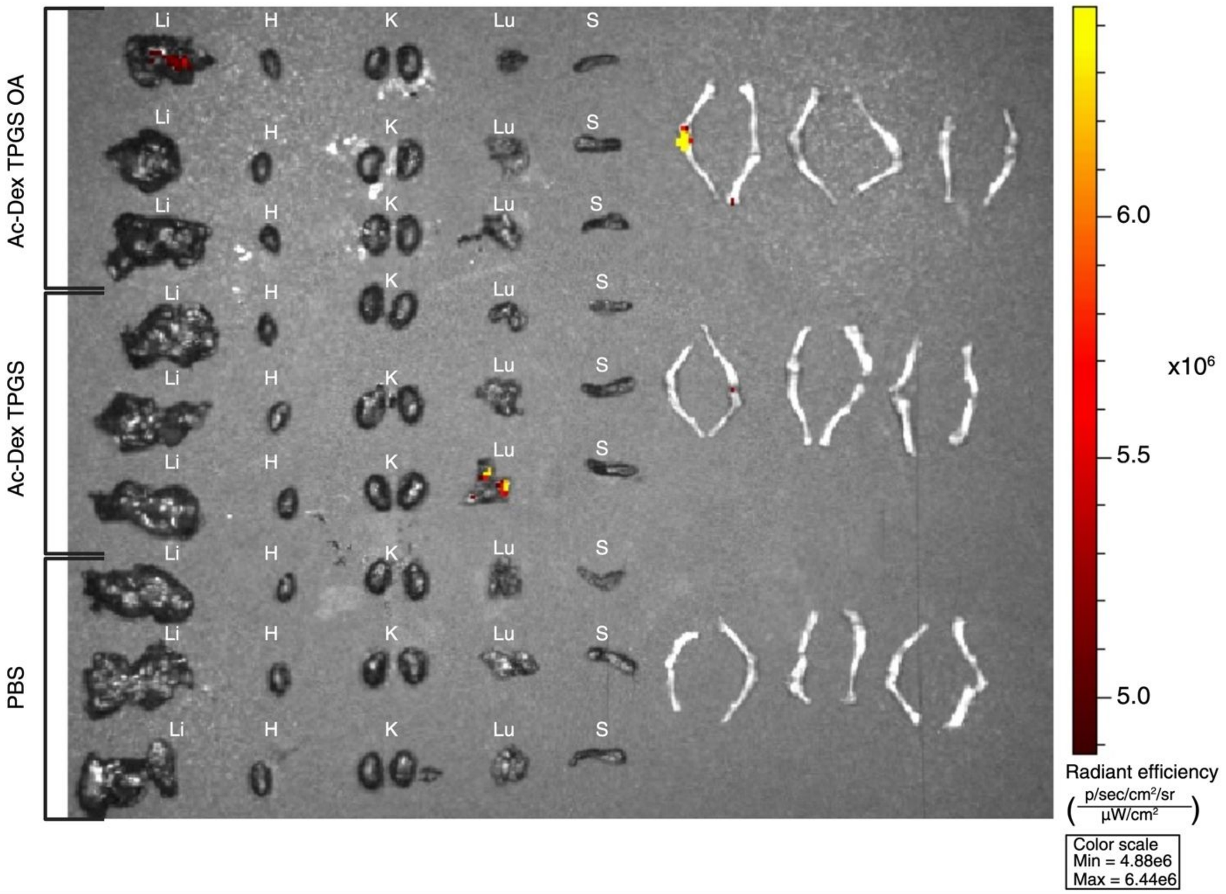

Figure S13 IVIS imaging comparing the accumulation of intravenously administered ICG loaded nanoparticles in the hindlimb (femur and tibia), liver (Li), heart (H), kidneys (K), lungs (Lu), and spleen (S). Imaging occurs 24 hours after the final dose of treatment that includes with Ac-Dex TPGS OA (*n* = 3), Ac-Dex TPGS *(n* = 3), and a PBS control *(n* = 3).

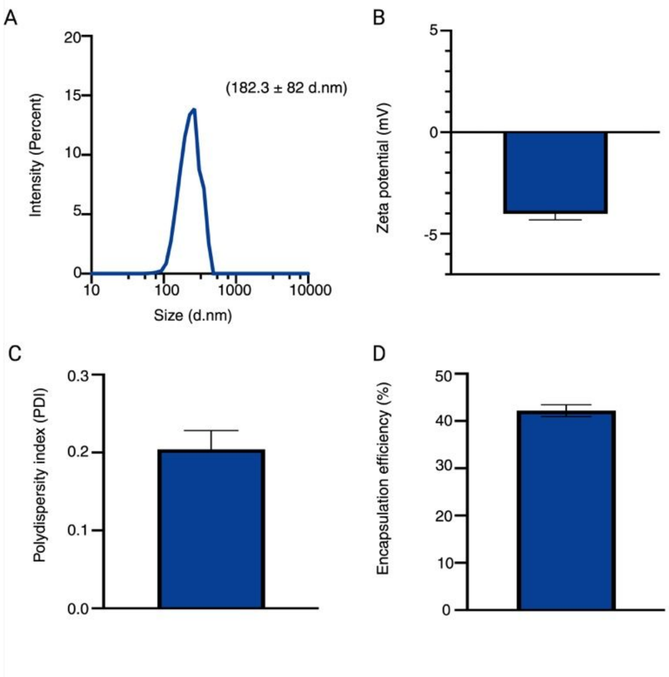

Figure S14 Physicochemical characteristics of Ac-Dex TPGS OA after encapsulating vincristine. (A) Size characterization is reported as intensity percentage recorded through dynamic light scattering (DLS). (B) The zeta potential was determined to measure the surface charge of the nanoparticle using electrophoretic light scattering (ELS). (C) The polydispersity index (PDI) to measure monodispersity was recorded through DLS. (D) Encapsulation efficiency (EE%) of vincristine was determined through nanodrop. Data are presented as mean ± s.d. (n= 3).

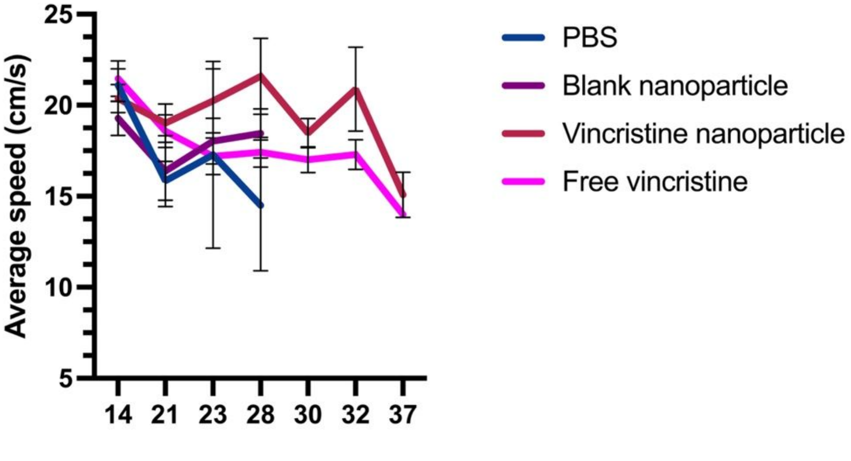

Figure S15 Open field testing demonstrated no significant difference in average speed. The average speed (cm/s) was recorded over 10-minute sessions for each time point. Treatments included PBS (n = 4), blank nanoparticles (n = 5), free vincristine (n = 10),

and vincristine loaded Ac-Dex TPGS OA nanoparticles (*n* = 10). Data are expressed as mean ± SEM.

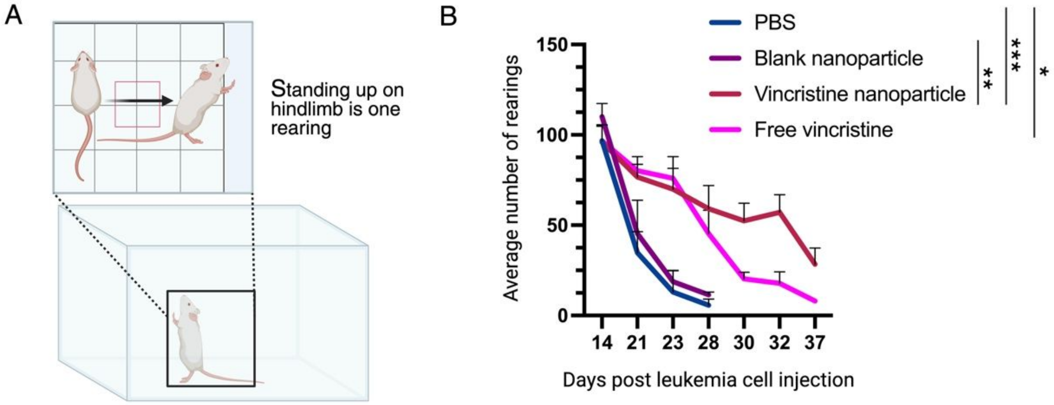

Figure S16 Open field testing demonstrates that vincristine based treatment groups retain the ability for rearing. (A) Schematic demonstrating that one rearing is defined as the mouse standing on its hindlimbs. (B) The average number of rearings was recorded over 10-minute sessions for each time point**.** Treatments included PBS (*n* = 4), blank nanoparticles (*n* = 5), free vincristine (*n* = 10), and vincristine loaded Ac-Dex TPGS OA nanoparticles (*n* = 10). Data are expressed as mean ± SEM. Statistical significance was determined by two-way ANOVA with Tukey's multiple comparison test. (**p* < 0.05; ***p* < 0.005; ****p* < 0.0005).

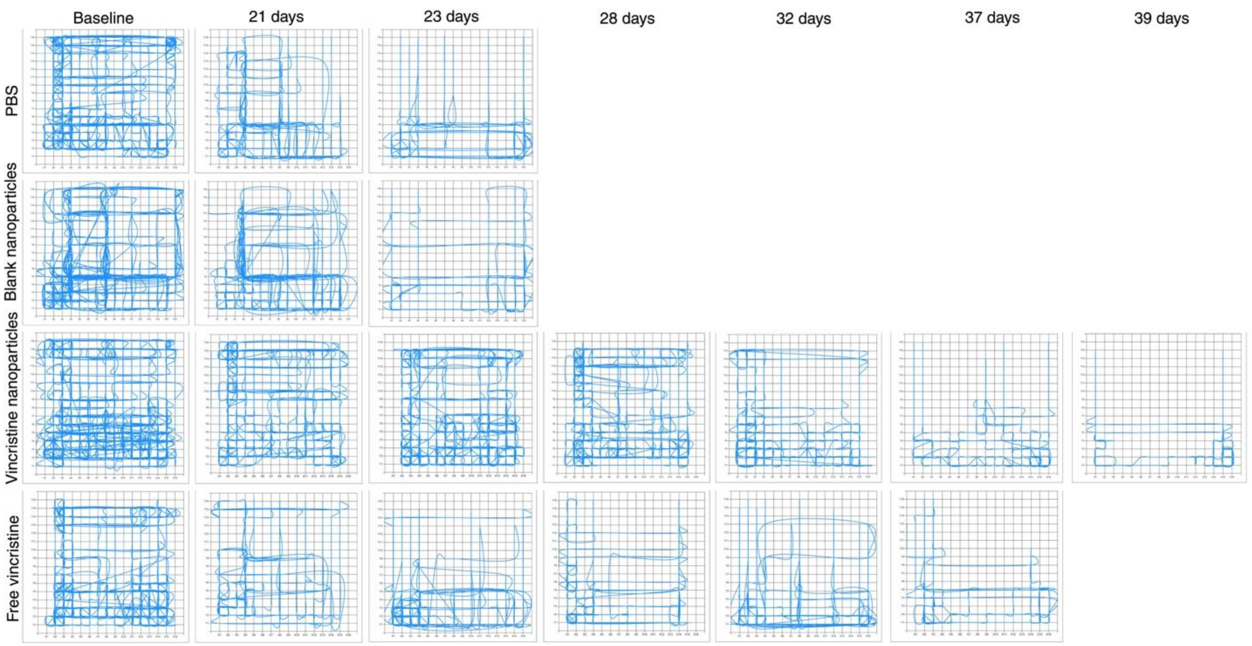

Figure S17 Representative tracking maps demonstrate preserved mobility in vincristine treated groups. Each map represents one mouse from each treatment group over the various time points during the 10-minute session. Reductions in movement coincide with advanced disease progression. Vincristine loaded nanoparticle groups visibly retained greater movement than free vincristine up to day 37.

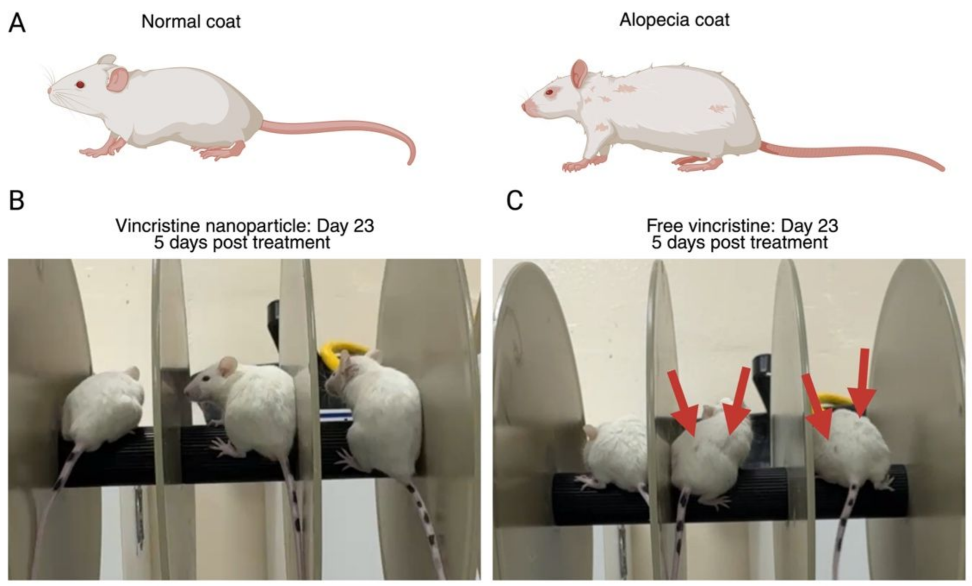

Figure S18 Visualization of NSG mice with well-groomed coats and preserved fur when treated with encapsulated vincristine within Ac-Dex TPGS OA nanoparticles. (A) Schematic illustrating a normal coat and a coat with alopecia in NSG mice. (B) Images of mice treated with vincristine loaded Ac-Dex TPGS OA nanoparticles 5 days after the final

treatment display preserved fur and a normal coat. (C) Images of mice treated with free vincristine 5 days after the final treatment display visible alopecia, indicated by the red arrows, which is consistent with systemic toxicity. Images were taken during rotarod testing using an iPhone 16 Pro Max under standard lighting.

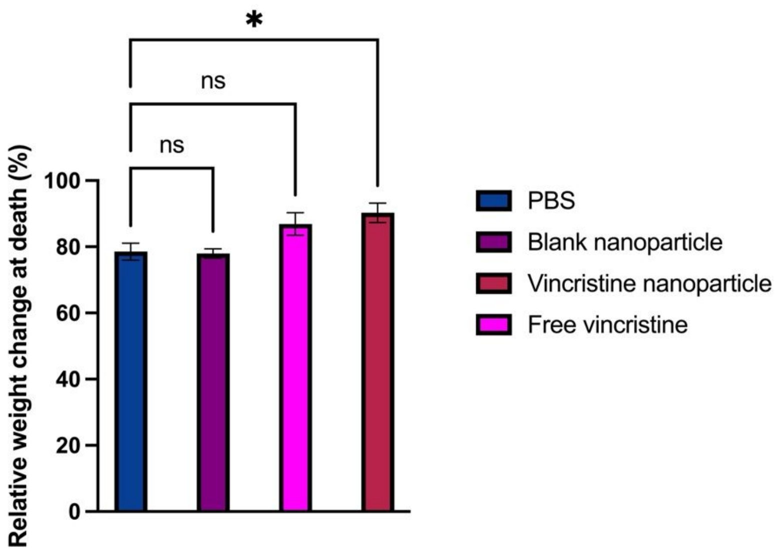

Figure S19 Vincristine loaded Ac-Dex TPGS OA nanoparticles have a higher relative body weight at death compared to controls and free vincristine. Treatments included PBS (*n* = 4), blank nanoparticles (*n* = 5), free vincristine (*n* = 10), and vincristine loaded Ac-Dex TPGS OA nanoparticles (*n* = 10). Data are presented as mean ± SEM. Statistical significance was determined by Brown-Forsythe one-way ANOVA with Dunnett's multiple comparisons test. (*$p < 0.05$; ns = not significant).

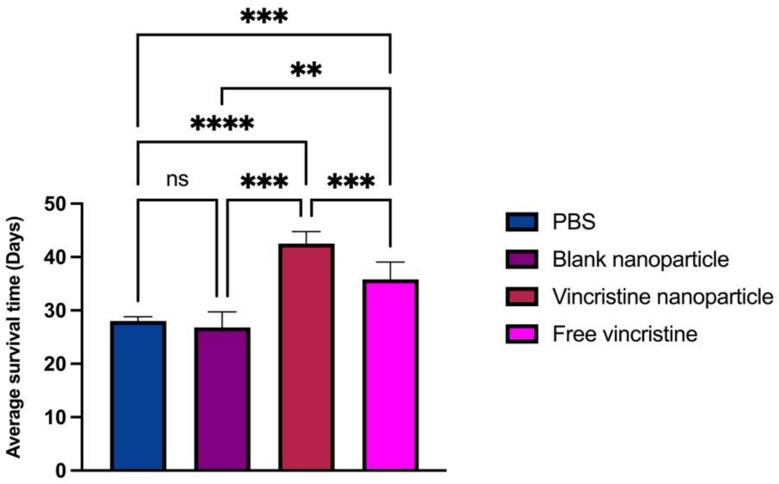

Figure S20 Mice treated with vincristine loaded nanoparticles survive significantly longer than other treatment groups. The average survival was calculated as days before the onset of euthanasia criteria following REH cell injection. Treatments included PBS (*n* =

4), blank nanoparticles (n = 5), free vincristine (n = 10), and vincristine loaded Ac-Dex TPGS OA nanoparticles (n = 10). Data are presented as mean ± SEM. Statistical significance was determined by Brown-Forsythe one-way ANOVA with Dunnett's multiple comparisons test. (**$p < 0.005$; ***$p < 0.0005$; ****$p < 0.0001$)
